# Neutrophil–macrophage crosstalk network in acute lung injury: feedback circuits linking cytokine storm and cell death

**DOI:** 10.3389/fcimb.2026.1864492

**Published:** 2026-07-07

**Authors:** Yu Ruoting, Lei Jinlin, Liu Yuhao, Fang Zemin, Gao Wei

**Affiliations:** 1 Department of Thoracic Surgery, Tianyou Hospital, Wuhan University of Science and Technology, Hubei, China; 2 Department of Cardiothoracic and Vascular Surgery, Tongji Hospital, Tongji Medical College, Huazhong University of Science and Technology, Hubei, China

**Keywords:** acute lung injury, cytokines, macrophages, neutrophils, PANoptosis

## Abstract

Neutrophils (NE) and macrophages (Mø) are canonical cellular components of the innate immune defense and are involved throughout the course of acute lung injury (ALI). The crosstalk between these two cell types is pivotal for elucidating the complex mechanisms underlying inflammation. This review focuses on the bidirectional interactions between NE and Mø mediated by cytokines and phagocytosis. In addition, emerging concepts, including exosomes and migrasomes, are discussed to further delineate the role of NE in this crosstalk network. Certain pro-inflammatory cytokines amplify inflammation through cascade effects, ultimately leading to a cytokine storm. Pro-inflammatory and anti-inflammatory processes are dynamically coordinated and diverge at key regulatory nodes, forming multiple positive and negative feedback loops that collectively establish the inflammatory network model of ALI. The terminal outcome of this network is cell death, encompassing apoptosis, necroptosis, pyroptosis, and PANoptosis. By integrating the crosstalk network with cell death pathways, a more comprehensive regulatory framework is constructed, enabling an in-depth exploration of the molecular mechanisms underlying ALI.

## 1 Introduction

Acute lung injury (ALI) is a common clinical diagnosis with diverse etiologies, including multiple trauma, infection, drugs, ventilator-induced injury, and connective tissue diseases. It is characterized by rapid disease progression and a prolonged clinical course (Liu et al., 2025). ALI is primarily manifested by hypoxemic respiratory failure, increased pulmonary capillary permeability, and pulmonary inflammation. Its underlying pathological mechanisms mainly involve cytokine participation, inflammatory cascade reactions, apoptosis, and necrosis (Zhu et al., 2023; Shan et al., 2024; Xu et al., 2024). In recent years, following the outbreak of coronavirus disease 2019 (COVID-19), both the incidence and mortality of ALI have increased (Liu et al., 2025). In clinical practice, management is largely supportive, including oxygen therapy, fluid resuscitation, and analgesia to alleviate pulmonary edema and respiratory dysfunction caused by injury (Goligher et al., 2021; Kakizaki et al., 2025), while etiological treatments targeting the underlying pathological mechanisms remain limited. The progression from lung tissue injury to ALI and, further, to acute respiratory distress syndrome (ARDS) represents a dynamic evolutionary process in which the crosstalk between neutrophils (NE) and macrophages (Mø) serves as a critical link and driving force. The primary distinction between ALI and ARDS lies in the PaO2/FiO2 ratio. In ALI, this value ranges from 200–300 mmHg, whereas in ARDS it is ≤200 mmHg (Mokra, 2020). In terms of disease severity, ALI is typically characterized by alveolar injury, hypoxemia, and pulmonary edema, whereas ARDS presents with diffuse lung tissue damage, severe hypoxemia, and respiratory failure, representing a more advanced and severe stage of ALI progression. NE and Mø are two key cell types that respond to inflammatory signals at the early stage of ALI and act as primary effectors in its pathogenesis. Their functions are broad, complex, and tightly interconnected, with a certain temporal sequence in their activation.

Reviews specifically focusing on the crosstalk between NE and Mø remain limited. Therefore, this review primarily uses lipopolysaccharide (LPS)-induced ALI as the experimental context to illustrate how key NE-derived products, including neutrophil extracellular traps (NETs), cytokines, and exosomes, mediate intercellular communication with Mø and subsequently regulate the polarization of distinct Mø phenotypes. As part of the downstream phase of the inflammatory response, Mø exhibit dual pro-inflammatory/anti-inflammatory functions that ultimately determine divergent inflammatory outcomes.

In addition, PANoptosis is a recently proposed concept observed in viral, bacterial, and fungal infections, as well as in inflammatory diseases. It is characterized by the concurrent occurrence of apoptosis, necroptosis, and pyroptosis (Wang et al., 2023), representing a complex crosstalk network among these cell death pathways. Given the conditions under which PANoptosis is triggered, existing reviews and studies have introduced this concept in the context of ALI (Chen et al., 2023; Yang et al., 2025). Accordingly, we integrate PANoptosis with classical modes of cell death to jointly examine the impact of NE and Mø death on inflammation.

In recent years, research in ALI has primarily focused on key regulatory molecules, genes, and potential therapeutic targets, with the ultimate goal of preventing or reversing disease progression. Within this context, we construct an inflammatory ALI model based on the NE–Mø crosstalk framework and, against the backdrop of disease progression, provide an in-depth description of the critical signaling pathways involved. This approach may, to some extent, facilitate the development of etiologically targeted therapies for ALI.

## 2 NE and Mø in ALI

NE, as classical effector cells of innate immunity, are recruited at the early stage of injury. Disruption of the alveolar–capillary barrier leads to pulmonary hematoma or edema. At this stage, dying alveolar epithelial cells and endothelial cells release inflammatory signals, to which NE rapidly responds and accumulates at the site of inflammation within 24 hours. These inflammatory signals can be classified, based on their molecular characteristics, into nucleic acids, proteins, ions, glycans, and metabolites (Ma et al., 2024). In ALI, they are primarily derived from damaged cellular components, including mitochondria, nuclei, endoplasmic reticulum, ATP, as well as cytokines secreted by NE themselves (Murao et al., 2021; Tian et al., 2025), and are collectively referred to as damage-associated molecular patterns (DAMPs) in the context of inflammation. In addition, LPS, commonly used to establish ALI models, is categorized as a pathogen-associated molecular pattern (PAMP) (Luo et al., 2025). It acts in concert with DAMPs on NE, promoting the phagocytosis of necrotic cells and cellular debris and mediating the resolution or progression of inflammation. However, when injury is severe and DAMPs derived from damaged cells and NE cannot be efficiently cleared and instead accumulate, further recruitment of NE is induced. Under such conditions, NE produces a wide array of cytokines and regulates Mø polarization through intercellular communication, thereby amplifying the inflammatory response. This stage corresponds to the “exudative phase” of ALI and represents the initiation of the NE–Mø inflammatory crosstalk network. The secretory modalities of NE include membrane-free release and extracellular vesicles. Among these, key components such as NETs released via membrane-free mechanisms, miRNAs carried within exosomes, and cytokines constitute critical pathways linking NE and Mø (Nakazawa et al., 2016; De Alessandris et al., 2019; Jiao et al., 2021).

**Figure 1:**
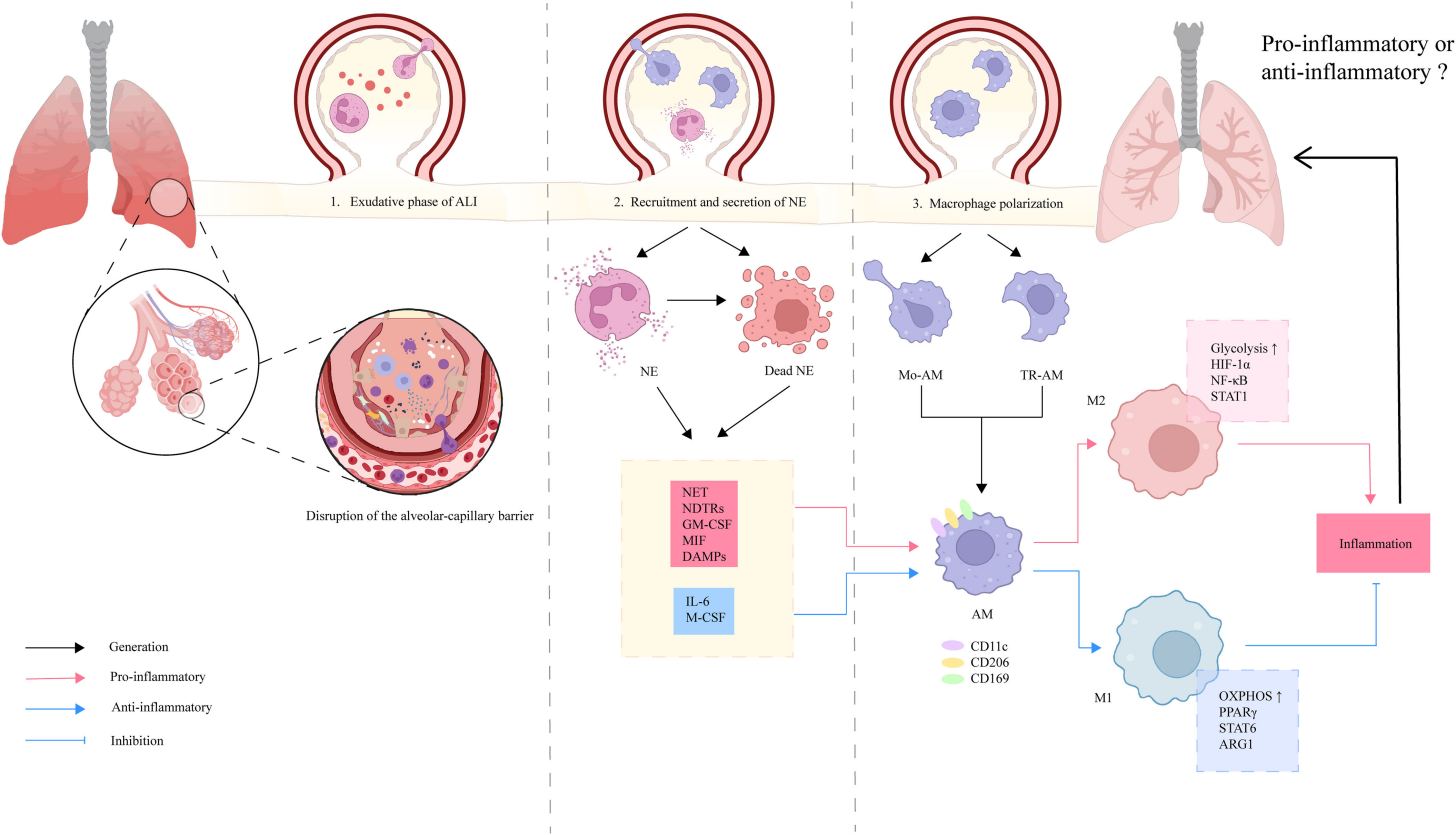
Key stages of ALI progression dominated by NE and Mø. In the early phase of ALI, NE extravasation initiates the inflammatory response, and its secretory function serves as a bridge linking Mø, thereby mediating the recruitment of pulmonary Mø and the differentiation of monocytes. Ultimately, these processes drive the inflammatory response toward a bifurcation in Mø phenotypic polarization.

At approximately 48 hours after injury, Mø exert their effects via two principal routes. First, peripheral monocytes extravasate into the lung and differentiate into Mø. Second, resident pulmonary macrophages (PM) are activated by inflammatory stimuli and act locally within the lung. Mø activated through both pathways can be regulated by NE via intercellular communication or phagocytic processes. PM comprises alveolar macrophage (AM) and interstitial macrophage (IM). Among these, AM represent a tissue-specific population that plays a predominant role in ALI. Compared with other Mø and circulating monocytes, AM are characterized by high expression of CD11c, CD206, and CD169, along with low expression of CD14 (Yu et al., 2016; Zhong et al., 2025). AM originates from two sources: tissue-resident AM (TR-AM) and monocyte-derived AM (Mo-AM). During lung injury, Mo-AM levels increase markedly, whereas in the later stages of recovery, Mo-AM declines substantially while TR-AM remains relatively stable (Janssen et al., 2011). Disruption of this balance may be associated with the inflammatory cascade observed in the later stages of ALI, and therapeutic strategies targeting the attenuation of Mo-AM activation have been explored (Ma et al., 2025). The recruitment and functional activation of both NE and Mø exhibit a certain temporal sequence, and their close interplay establishes key regulatory pathways during the early phase of inflammation. (**Figure 1**).

In addition, Mø polarization plays a important role in the progression of inflammation during early lung injury. Accumulating evidence has challenged the traditional view that M1- and M2-polarized Mø possess strictly opposing functions (Xue et al., 2014). Instead, Mø polarization is now recognized as a continuous, dynamic process that generates a broad spectrum of phenotypic states. M1 and M2 represent the two extremes of this polarization continuum and reflect the predominant functional orientations of Mø in inflammation. The polarization process involves highly complex signaling pathways, with metabolic reprogramming as the fundamental driving force (Viola et al., 2019). M1 polarization is characterized by a predominance of glycolysis, interruption of the tricarboxylic acid (TCA) cycle with accumulation of citrate, succinate, and itaconate, and engagement of the inducible nitric oxide synthase (iNOS) pathway in amino acid metabolism. Key regulatory molecules associated with this phenotype include hypoxia-inducible factor-1α (HIF-1α), nuclear factor-κB (NF-κB), and signal transducer and activator of transcription 1 (STAT1), which collectively mediate the production of pro-inflammatory cytokines and amplify inflammatory responses. In contrast, M2 polarization is primarily dependent on oxidative phosphorylation (OXPHOS), features an intact TCA cycle, and utilizes the arginase-1 (ARG1) pathway in amino acid metabolism. This phenotype is regulated by molecules such as peroxisome proliferator-activated receptor γ (PPARγ), STAT6, and peroxisome proliferator-activated receptor gamma coactivator-1β (PGC1β), constituting classical anti-inflammatory pathways that promote the expression of anti-inflammatory mediators and enhance the phagocytic capacity of Mø. Recent studies have demonstrated that activation of pro-inflammatory Mø does not necessarily require suppression of OXPHOS (Ball et al., 2025), further highlighting the diversity and complexity of metabolic pathways underlying Mø activation. Thus, pro-inflammatory and anti-inflammatory Mø phenotypes do not rely on a uniform metabolic program.

ALI is a heterogeneous syndrome characterized by diffuse alveolar inflammatory infiltration, thickening of the alveolar septa, and hyaline membrane formation. However, no currently available animal model can fully recapitulate all of these pathological features (Matute-Bello et al., 2011), which remains one of the major barriers limiting the translation of experimental findings into clinical practice. Among existing models, LPS-induced ALI is the most commonly used approach and is widely adopted because of its high reproducibility (Tan et al., 2024). Nevertheless, this model has several limitations. Variations in LPS purity and composition may influence the downstream signaling pathways activated, and the model fails to adequately mimic sterile inflammation. In contrast, pulmonary contusion models better reflect sterile inflammatory injury but often suffer from poor reproducibility. Furthermore, although the traditional M1/M2 classification of Mø has been recognized as an oversimplification, most ALI studies continue to rely on this framework. Emerging evidence indicates that tissue-resident alveolar macrophages (TR-AMs) and monocyte-derived alveolar macrophages (Mo-AMs) exhibit distinct functions during ALI (Mould et al., 2017) Therefore, integrating single-cell sequencing data with functional analyses of Mø across different ALI models may facilitate a more comprehensive understanding of the underlying mechanisms and advance mechanistic research in the field. 2.1 Initiation: membrane-free secretion of NE.

In the membrane-free secretion mode, NE-derived products are directly released without encapsulation by the plasma membrane. Among these, NETs have attracted considerable attention as classical pro-inflammatory mediators. Existing studies have highlighted the antimicrobial functions of NETs in a variety of disease settings (Brinkmann et al., 2004). However, in most studies of ALI, NETs are primarily associated with the amplification of inflammation and the exacerbation of lung tissue injury, with their ability to trigger inflammatory cascades appearing to play a dominant role. Among these effects, the regulation of Mø polarization by NETs represents a critical mechanism (Li et al., 2021). The following section summarizes NET components that have been experimentally validated in ALI and discusses the mechanisms by which these components promote the secretion of key cytokines, IL-1β and TNF-α, as well as the corresponding responses of Mø. Together, these interactions form a NET–IL-1β/TNF-α pro-inflammatory circuit that plays a critical role in both early pathogen clearance and the subsequent amplification of inflammatory cascades during ALI.NETs are composed of chromatin decorated with antimicrobial proteins, forming a web-like structure. In ALI, they are directly released by NE to facilitate pathogen clearance and enhance the phagocytic capacity of NE. However, when NET activation is excessive and accumulates locally without timely clearance, both granule proteins and NETs themselves can act as DAMPs on Mø, mediating Mø chemotaxis, secretion, and phenotypic differentiation—predominantly toward M1 polarization (Qin et al., 2019; Scozzi et al., 2022; Jiang et al., 2024).

The antimicrobial protein composition of NETs varies depending on the mode of activation. Among these, key components that exhibit prominent crosstalk with Mø include myeloperoxidase (MPO), LL-37 and High-mobility group box 1 (HMGB1). LL-37, a broadly active antimicrobial peptide, can interact with multiple receptor families, including G protein–coupled receptors (GPCRs), receptor tyrosine kinases (RTKs), ligand-gated ion channels (LGICs), and Toll-like receptors (TLRs), thereby triggering diverse downstream biological effects. The P2X7 receptor (P2X7R) is involved in Mø uptake of LL-37. As a K^+^-gated ion channel, P2X7R is functionally linked to the assembly of the NLRP3 inflammasome. This process is accompanied by substantial K^+^ efflux, which promotes the maturation and release of IL-1β and IL-18 (Kahlenberg et al., 2013). In contrast, in LPS-induced murine models, LL-37 exerts protective effects (Gao et al., 2025), enhancing autophagy by inhibiting ZBP1 gene expression. Current prevailing evidence suggests that inhibition of P2X7R can reduce the secretion of pro-inflammatory cytokines by Mø and attenuate oxidative stress in lung tissue (Ozkanlar et al., 2025).

At 8 hours after lung injury, MPO levels in lung tissue are significantly elevated, and at the same time point (8 hours), TLR4 protein expression in lung tissue reaches its peak (Wu et al., 2012). This finding may suggest that, in the context of lung injury, MPO—as a marker of NETs—reflects both the severity of lung damage and NE activity. MPO can interact with immune receptors expressed on Mø (such as TLR4), thereby enhancing their functional responses and markedly upregulating pro-inflammatory cytokine production. This interaction drives AM toward an M1-like polarization state (Grattendick et al., 2002; Taylor et al., 2005), inducing the generation of reactive oxygen intermediates (ROI) and tumor necrosis factor-α (TNF-α).HMGB1 is a nuclear protein that is ubiquitously distributed in the nucleus and cytoplasm of various cell types under physiological conditions. During NETosis, HMGB1 is released together with NETs. HMGB1 can bind to the receptor for advanced glycation end products (RAGE) on Mø and induce Mø pyroptosis in a caspase-1-dependent manner (Chen et al., 2018). This pathway has been further characterized in ALI associated with multiple organ dysfunction syndrome (MODS), where HMGB1 promotes IL-1β, IL-6 production, and AM autophagy through upregulation of NOD2, a cytosolic pattern-recognition receptor (Wen et al., 2014). Although AM autophagy is generally considered an anti-inflammatory process, this study demonstrated that supplementation with PMNs counteracted its protective effects. This phenomenon was associated with NADPH oxidase activity in PMNs. Collectively, these findings suggest that IL-1β secretion by M1 Mø is a key driver of inflammatory amplification, whereas the recruitment and accumulation of NE provide an essential basis for sustaining the inflammatory response.

In addition, NETs themselves can promote the polarization of Mø toward the M1 phenotype, leading to increased secretion of Cathepsin C (CTSC) and the formation of a “positive feedback amplification loop” in pulmonary inflammation (Yu et al., 2024), is significantly positively correlated with lung tissue injury. As a marker of NETosis, Cathepsins comprise a group of distinct intracellular proteases. Among them, CTSC is a cysteine protease belonging to the papain-like cysteine peptidase family. It is primarily localized in lysosomes and exerts pro-inflammatory and antimicrobial effects in innate immunity by activating key proteases, including NE elastase, cathepsin G, and proteinase 3. CTSC is present in both Mø and NE and can be directly released into the extracellular space to mediate intercellular communication. Mø-derived CTSC can activate p38 MAPK signaling in NE, subsequently stimulating NADPH oxidase and promoting the generation of reactive oxygen species (ROS), thereby reinforcing NETosis through a positive feedback mechanism. Meanwhile, CTSC-mediated activation of surface PR3 (mPR3) on NE can upregulate IL-1β secretion. This establishes a PR3–IL-1β–p38 signaling axis that further sustains the positive feedback loop of NETosis. This pro-inflammatory circuit has been well validated in ALI (Wu et al., 2025), The positive feedback loop involving CTSC secretion by NE may represent a critical event in the amplification of inflammation during ALI. As a highly active protease, CTSC plays important roles in various ALI models, including LPS-induced injury, ischemia–reperfusion injury, mechanical ventilation-induced lung injury, and viral infection (Korkmaz et al., 2020; Seren et al., 2021; Yu et al., 2024).

**Figure 2:**
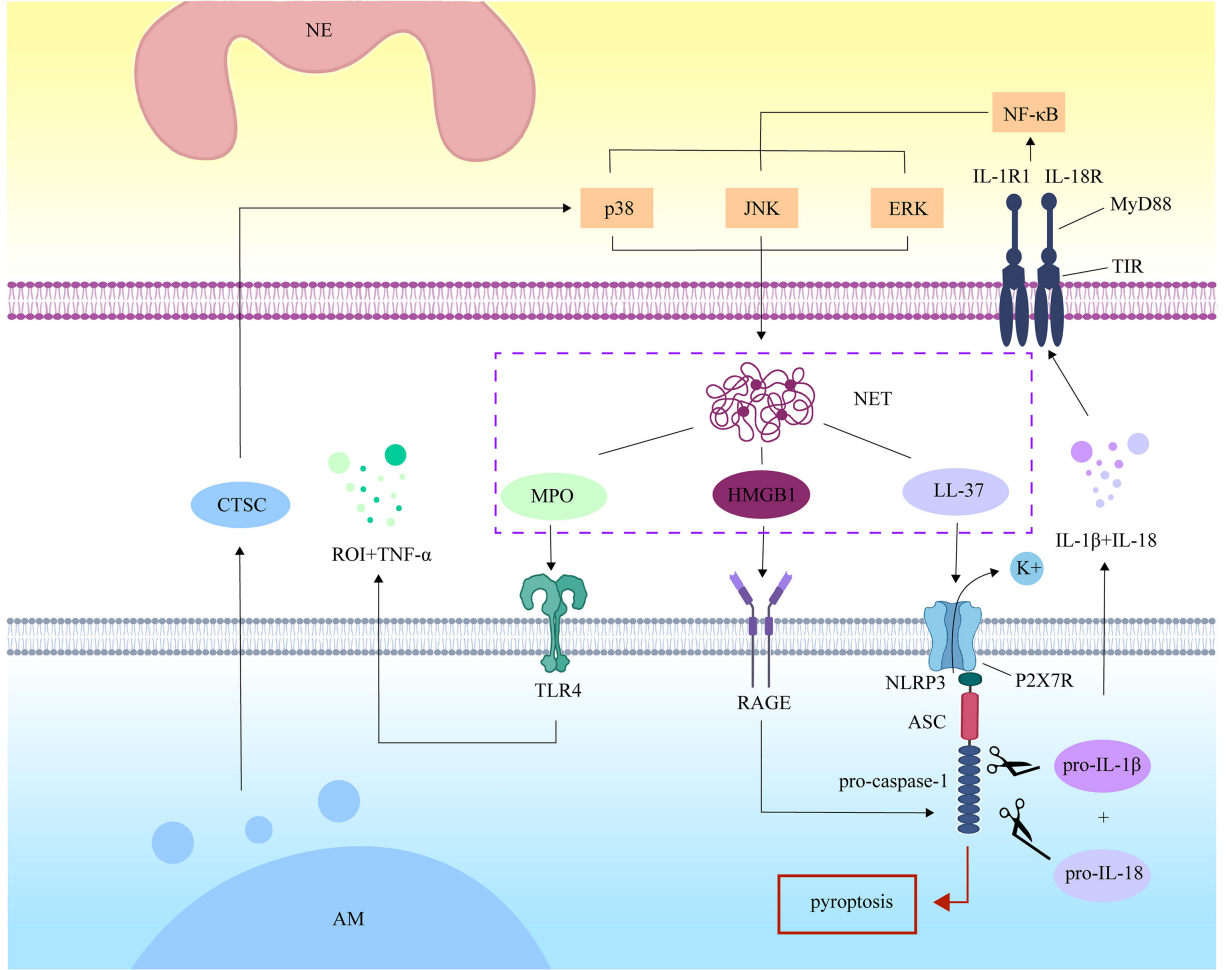
NET-mediated NE–Mø micro inflammatory crosstalk network. The core components of NETs (LL-37, HMGB1 and MPO), both exert pro-inflammatory effects. They act on macrophage surface receptors, activating the release of Mø-derived CTSC, TNF-α, ROI, and IL-1β/IL-18, which in turn feed back upstream to promote NET formation. Collectively, these interactions establish a complete pro-inflammatory circuit between NE and Mø.

IL-1β, IL-18, and TNF-α released from Mø subsequently bind to IL-1R1, IL-18R, and TNFR1/2 on NE, respectively. The downstream signaling pathways of these receptors converge on the activation of NF-κB, a key transcription factor that promotes NET formation. This returns the signaling cascade to the upstream stage of the NET–IL-1β/TNF-α circuit, further amplifying the pro-inflammatory functions of NE, enhancing their phagocytic and pathogen-clearing capacities, while simultaneously exacerbating pulmonary inflammation and tissue injury (**Figure 2**). In addition, neutrophil elastase has been shown to promote the release of macrophage extracellular traps (METs). Based on current evidence (Doster et al., 2017; Kummarapurugu et al., 2021), we propose that the downstream regulatory network of the NET–IL-1β/TNF-α circuit remains incompletely characterized and may also involve feedback from METs to NE. However, the specific effects of METs in ALI have not yet been adequately elucidated. Therefore, further studies are required to define the contribution of METs and to refine our understanding of the reciprocal interactions between these two cell types.In summary, NETs primarily promote inflammation during ALI progression. In the later stages of inflammation, their role in mediating tissue injury surpasses their auxiliary function in supporting NE phagocytosis. NETs not only drive Mø polarization toward the M1 phenotype but also show a positive correlation with pro-inflammatory cytokine levels, including IL-6 and TNF-α (Song et al., 2019). Through their complex composition, NETs engage in intensive interactions with both NE and AM, thereby amplifying the inflammatory response. NETs also contain numerous biologically active proteins, such as PR3 and Cat G, whose specific functions within NETs have not yet been fully elucidated. The pro-inflammatory effects of LL-37 on Mø have been demonstrated in ALI. However, when considered alongside existing experimental findings, NET-derived LL-37 appears to exert opposing effects on Mø and NE themselves, suggesting that its functions may vary across different stages of inflammation. The downstream signaling pathways associated with NETs are highly complex and exhibit distinct patterns in different types of ALI (Barnes et al., 2020). Therefore, more precise characterization of NETs across diverse ALI models is needed. In addition, the pro-inflammatory positive feedback loop downstream of CTSC has been validated in multiple studies, indicating that this mechanism may be broadly applicable to various forms of ALI. As such, CTSC-related pathways possess substantial translational and clinical potential.2.2 NE-derived extracellular vesicles.

Extracellular vesicles (EVs) are a collective term for membrane-bound particles naturally released by cells. Those derived from NE include NE-derived microvesicles (NDMVs), neutrophil-derived trails (NDTRs), and neutrophil-derived structures (ENDS) (Théry et al., 2018; Youn et al., 2021; Marki and Ley, 2022), all of which contain intracellular components and bioactive molecules from NE. Each of these EV subtypes can exert regulatory effects on Mø.

NDMVs are generated when NE receives stimulation from bacteria, cytokines, or complement, through outward budding of the plasma membrane, and are shed during inflammation or apoptosis. They possess a double-membrane structure, and their surface is decorated with phosphatidylserine (PS) and adhesion molecules. It contains not only organelles but also a variety of components derived from their parental NE, including adhesion molecules, Fc and complement receptors, NE granules, cytosolic proteins (e.g., S100A8), and microRNAs. These vesicles are generated when NE arrives at sites of injury in response to inflammatory mediators such as pro-inflammatory cytokines, complement components, and nitric oxide (NO) (Stein and Luzio, 1991; Nolan et al., 2008; Timár et al., 2013). NDMVs can produce ROS upon stimulation with NO, Ca²^+^ ionophores, and phorbol 12-myristate 13-acetate (PMA), and generate leukotriene B4 (LTB4) in response to arachidonic acid and Ca²^+^ ionophores (Dalli et al., 2013). LTB4 acts on BLT1, which is expressed on Mø, and, through GPCR coupling, promotes phagocytosis and NO production. Notably, the presence of NDMVs has been shown to suppress the expression of pro-inflammatory cytokines in Mø while enhancing the secretion of anti-inflammatory cytokines such as TGF-β and IL-10. This effect is associated with the downregulation of zymosan A (ZymA)-induced signaling and the involvement of the MerTK pathway (Eken et al., 2010; Eken et al., 2013). However, *in vitro* studies have also demonstrated that NE-derived EVs can impair the anti-inflammatory secretory capacity of Mø, rendering them “unresponsive” to secondary stimulation following EV exposure. In a LPS-induced cytokine storm syndrome (CSS) model, NDMVs deliver miR-27a-3p to suppress Suclg1 expression in Mø, leading to the accumulation of itaconate catabolism intermediates and thereby promoting Mø polarization toward the M2 phenotype (Kang et al., 2024). In recent years, EVs have been increasingly investigated as key targets in ALI research. In summary, NDMVs generally function as anti-inflammatory mediators in the circulation. However, in ALI models, EVs often appear to exacerbate inflammatory responses, which may be attributed to ALI heterogeneity and differences in their molecular cargo. For instance, Jiao et al. demonstrated that NE-derived EVs containing miR-30d-5p promote Mø polarization toward the M1 phenotype and identified a TNF-α–EVs–NF-κB pro-inflammatory signaling axis *in vitro*. They further identified suppressor of cytokine signaling 1 (SOCS-1) and sirtuin 1 (SIRT1) as downstream targets of miR-30d-5p, and confirmed that inhibition of miR-30d-5p attenuates M1 polarization of Mø and alleviates lung tissue injury (Jiao et al., 2021). Experimental evidence also indicates that the miRNA profiles carried by EVs in the airway vary depending on the etiology of ALI (Nakatsutsumi et al., 2025). Nevertheless, studies specifically addressing the effects of NDMVs on Mø remain limited, and the precise composition and mechanistic roles of NDMV cargo require further elucidation.

Studies have shown that NDTRs are membrane-bound remnants generated during NE migration toward sites of inflammation. NDTRs share similar structural and functional characteristics with NDMVs, and both form stable oval-shaped structures. However, their biogenesis differs, as NDTR formation is dependent on integrins, whereas NDMVs are generated via the PI3K pathway. In addition, differential surface marker expression distinguishes the two populations, with NDMVs highly expressing CD16 and NDTRs exhibiting high levels of PSGL-1. MCP-1 contained within NDTRs enhances monocyte chemotaxis, and M0 Mø cultured in an NDTR-rich environment exhibit increased expression of pro-inflammatory markers, such as inducible nitric oxide synthase (iNOS), IL-12, and TNF-α. This effect may be associated with the specific miRNA cargo of NDTRs, including miR-1260a, miR-1285-5p, miR-4454, and miR-7975 (Youn et al., 2021). Compared with NDMVs, NDTRs are more associated with M1 Mø polarization and migration. ENDS, in contrast to the two aforementioned extracellular vesicles, exhibit an elongated morphology and do not contain endoplasmic reticulum or DNA. Their surface can also express PS, and they are significantly upregulated in the blood of patients with sepsis. They are significantly upregulated in septic mouse models and release the S100A8–S100A9 complex (Marki et al., 2020). S100A9 has been shown, both *in vivo* and *in vitro*, to exacerbate lung tissue injury and to promote Mø polarization toward the M1 phenotype via the TLR4–MyD88–NF-κB signaling pathway, promote pyroptosis of Mø (Gong et al., 2024). S100A8 exhibits similar functional properties and signaling pathways (Luo et al., 2024). Due to the limited number of available studies, the specific roles of NDTRs and ENDS in ALI have not yet been fully elucidated, current investigations into their regulatory mechanisms are still largely derived from classical inflammatory pathways. Their specific regulatory molecules remain under exploration, and the difficulty of studying these vesicles may stem from the unique spatial and temporal characteristics of their generation. But accumulating experimental evidence, along with the fact that both belong to the NDMV family and are generated during NE migration, These findings raise the possibility that specific therapeutic targets may be exploited to attenuate inflammatory cell infiltration into lung tissue during the early stages of inflammation.

### 2.3 NE-derived cytokines

The secretion of cytokines plays a regulatory role in Mø recruitment and phenotypic polarization and is often a prerequisite for the development of a cytokine storm in ALI. Key NE-derived cytokines include M-CSF, GM-CSF, IL-6, and Mø migration inhibitory factor (MIF) (**Table 1**).

**Table 1:** Effects of NE-derived cytokines on Mø.

Cytokine	Receptor on macrophages	Functional effect	Mechanistic notes	Reference
IL-6	IL-6Rα / gp130	bidirectional regulation of inflammation	Promotes M2 polarization via STAT6; inhibits pyroptosis	(Mauer et al., 2014; Gou et al., 2022)
M-CSF	CSF1R (CD115)	Anti-inflammatory	Induces M2 differentiation; enhances phagocytosis and tissue repair	(Chen et al., 2021)
GM-CSF	GM-CSFR (CD116)	Pro-inflammatory	Activates JAK2–STAT5 signaling; promotes M1 polarization and cytokine release	(Paine et al., 2001; Martinez and Gordon, 2014)
MIF	CXCR2	Pro-inflammatory	Enhances macrophage recruitment and cytokine production	(Tilstam et al., 2021)

M-CSF and GM-CSF act on CD115 and CD116, respectively, expressed on the surface of Mø (Waldo et al., 2008). M-CSF is constitutively produced under homeostatic conditions and mediates the differentiation of monocytes into Mø, serving as a key cytokine driving Mø development. Upon binding to CD115, M-CSF activates multiple downstream signaling pathways, including PI3K, ERK, and TNF-related cascades, thereby inducing Mø polarization toward the M2 phenotype. This is characterized by enhanced phagocytic activity and increased secretion of anti-inflammatory cytokines such as IL-10, MCP-1, and IL-39 (Hamilton, 2008; Mabbott et al., 2010; Ushach et al., 2015; Chen et al., 2021). In contrast, GM-CSF is expressed at low levels under physiological conditions but is markedly upregulated in inflammatory environments. During lung injury, GM-CSF recruits Mø and promotes the maturation of AM; GM-CSF deficiency in lung tissue is associated with increased susceptibility to infection (Riopel et al., 2001). GM-CSF signals through CD116 and, in contrast to M-CSF, its expression is induced by pro-inflammatory cytokines. It activates the canonical JAK2–STAT5 pathway, promoting robust recruitment of Mø (Paine et al., 2001; Martinez and Gordon, 2014). This pathway is closely associated with M1 polarization of Mø.

IL-6, a cytokine produced by multiple immune cell types, which serves as a key biomarker of inflammatory activity in ALI (Knöferl et al., 2003). Its receptor complex consists of the IL-6 binding receptor (IL-6Rα) and the signal-transducing component gp130, both of which are expressed on NE and Mø. The ratio of its soluble receptor (sIL-6R) to the antagonist soluble glycoprotein 130 (sgp130) correlates with the severity of lung tissue injury (Chepurnova et al., 2018). Previous studies have demonstrated that IL-6 can directly induce IL-4 receptor expression (Mauer et al., 2014), thereby activating the downstream STAT6 pathway. By upregulating Arg1 expression (a surface marker of M2), IL-6 serves as an important initiating factor in the negative feedback regulation of inflammation. However, this protective effect has thus far been observed only in obese patients with sepsis, and whether it can be extended to other ALI models remains controversial. We further speculate that IL-6 may promote IL-4 production, thereby generating a distinct Mø phenotype characterized by high IL-4 expression that plays an important role in ALI-associated inflammation (Murray et al., 2014). Nevertheless, the efficacy of this protective mechanism may be influenced by multiple factors, including disease etiology and host physiological status. In addition, IL-6 can reduce pyroptosis of AM during inflammation through the caspase-1–GSDMD–IL-1β axis (Gou et al., 2022), This study suggests that, in infection-induced ALI, IL-6 most likely functions as a protective cytokine. It may exert antimicrobial effects through a Mø-dependent mechanism, enhances Mø survival to sustain these protective functions, and reduces the release of pro-inflammatory mediators generated during pyroptosis. Protective effects of IL-6 have been demonstrated in both hyperoxia-induced and infection-induced ALI models (Kolliputi and Waxman, 2009; Woods et al., 2015). However, in the early stages of transplantation-related ALI and ALI secondary to injury in other organs, IL-6 levels remain positively correlated with the severity of lung injury (Zhang et al., 2013; Varelias et al., 2015). Excessive production of IL-6 is commonly associated with severe inflammation and may provide a mechanistic link between ALI and other forms of organ injury, such as hepatic or pancreatic damage, as well as sepsis (Peng et al., 2024; Liu et al., 2025). Further investigation of IL-6-driven inflammatory cascades may facilitate the identification of therapeutic targets for specific subgroups of patients with ALI.

MIF is passively released when NE undergoes apoptosis and is not efficiently cleared (Roth et al., 2015), and its expression is induced through interaction with hypoxia-inducible factor-1α (HIF-1α). This characteristic may render MIF a potential biomarker of tissue injury. MIF acts on CXCR2 to promote directed migration of monocytes. It also induces migration arrest in Mø (Tilstam et al., 2021), leading to Mø accumulation and production of pro-inflammatory cytokines. These seemingly contradictory effects may be associated with CXCR2 desensitization (Bernhagen et al., 2007). In ALI mouse models, MIF secretion upregulates the mRNA expression of inflammatory cytokines in multiple immune cell types, including Mø (such as TNF-α, IFN-γ, IL-1β, and IL-6). MIF promotes the recruitment of both NE and Mø into the lungs without affecting circulating leukocyte counts, suggesting that MIF may exert predominantly local effects within the pulmonary compartment (Spiller et al., 2025). This localized activity may provide a novel perspective for the treatment of ALI, supporting the development of targeted therapeutic strategies centered on the lung.

NE, through cytokines and endogenous inflammatory mediators, plays an important role in initiating the classical inflammatory activation in ALI. NE-derived cytokines and EVs recruit Mø from the peripheral circulation to the lung while simultaneously activating alveolar Mø. Sustained NE infiltration is a hallmark of the early inflammatory response in ALI, which may contribute to increased vascular permeability and the development of pulmonary edema. At this stage, NET release contributes to the formation of pulmonary microthrombi within the lung microvasculature. These pathological changes are associated with reduced lung compliance, impaired gas diffusion, ventilation/perfusion (V/Q) mismatch, and intrapulmonary shunting (Coste et al., 2022). Collectively, these abnormalities may promote severe hypoxemia and subsequent tissue hypoperfusion. Overall, NE primarily drives the positive feedback of inflammation during the progression of ALI, amplifying the inflammatory cascade.

## 3 Response: Mø-mediated inflammatory feedback circuits

Recruited to sites of injury, Mø phagocytose cellular debris derived from dead cells while also secreting cytokines that modulate NE activity (**Table 2**). Attracted by dying cells and inflammatory mediators, Mø undergo differentiation upon arrival at the injury site. Among them, AM, being in closest proximity, are the first to become activated and polarize toward the M1 phenotype, releasing pro-inflammatory cytokines (Vergadi et al., 2014; Bhatia et al., 2012; Pichert et al., 2012). This process further recruits NE to transmigrate across the vascular endothelium and epithelial barrier into the inflamed tissue, thereby amplifying the inflammatory response. Excessive M1 activation, together with protein deposition resulting from immune responses, can induce additional alveolar epithelial cell necrosis, aggravate pulmonary edema, and promote alveolar collapse leading to atelectasis. Moreover, necrosis of alveolar epithelial cells reduces pulmonary surfactant (PS) secretion, resulting in severe respiratory dysfunction that may become life-threatening. Through these mechanisms, a pro-inflammatory positive feedback loop is established between NE and Mø. During the resolution phase of inflammation, Mø that have phagocytosed apoptotic cells undergo a phenotypic shift toward the M2 state. These M2 primarily function to clear apoptotic cells and secrete anti-inflammatory cytokines, which suppress further inflammatory progression (Cheng et al., 2021; Suleimanov et al., 2024), reduce the production of inflammatory mediators, and promote the restoration of pulmonary function. This constitutes a protective negative feedback loop. Together, these two opposing circuits determine the divergent outcomes of inflammation.

**Table 2:** Effects of Mø-derived cytokines on NE.

Source cell	Cytokine	Receptor on neutrophils	Functional effect	Mechanistic notes	Reference
M1	TNF-α	TNFR1 / TNFR2	bidirectional regulation of inflammation	Activates NF-κB and ROS production; regulates neutrophil survival/apoptosis	(Volk et al., 2011; Chen et al., 2021)
M1	IL-6	IL-6R / gp130	bidirectional regulation of inflammation	IL-6 induces IL-8 expression and activates STAT3; in contrast, the IL-6/sIL-6R complex inhibits NE chemotaxis and promotes macrophage recruitment.	(Hurst et al., 2001; Fielding et al., 2008; Wright et al., 2014)
M1	IL-1β / IL-18	IL-1R1 / IL-18R	Pro-inflammatory	Induces NET formation and neutrophil recruitment	(Kahlenberg et al., 2013)
M1	IL-8 (CXCL8)	CXCR1 / CXCR2	Pro-inflammatory	Potent neutrophil chemoattractant; enhances respiratory burst	(Krupa et al., 2004; Fudala et al., 2007)
M1	CXCL1 / CXCL2	CXCR2	Pro-inflammatory	Promotes neutrophil recruitment and survival	(Reutershan and Ley, 2004; Zhou et al., 2024)
M2	IL-10	IL-10R	Anti-inflammatory	Activates STAT3; suppresses pro-inflammatory cytokine production	(Ouyang and O’Garra, 2019)
M2	TGF-β	TGFβR (TβRI/TβRII)	bidirectional regulation of inflammation	Suppresses ROS and degranulation; may promote migration/ inhibit NE apoptosis	(Ear et al., 2010; Guven et al., 2013)

### 3.1 Secretory functions of distinct Mø phenotypes

Pro-inflammatory cytokines secreted by M1 include TNF-α, IL-6, IL-1β, IL-8, and CXCL-1/2. Among these, TNF-α, as a classical inflammatory cytokine, is markedly upregulated in ALI models characterized by abundant M1 (Zhao et al., 2024). TNF-α binds to TNF-R1 or TNF-R2 expressed on NE and exerts distinct effects through two signaling pathways. In the pro-inflammatory pathway, TNF-R1–associated complexes (or TNF-R2 itself) activating the NADPH oxidase and NF-κB pathways. NADPH oxidase leads to the production of ROS, thereby exacerbates surrounding tissue injury (Volk et al., 2011), while also conferring resistance to NE apoptosis. Experimental evidence suggests that FoxO3a may serve as an important transcription factor downstream of TNF-α in ALI, and it functions in parallel with JNK as two independent signaling pathways. In ALI models, knockdown of FoxO3a or pharmacological inhibition using SP600125 (a JNK inhibitor) can reverse the pro-inflammatory effects mediated by both pathways, thereby promoting NE apoptosis and protecting lung tissue (Chen et al., 2021). In contrast, only TNF-R1 can mediate anti-inflammatory signaling pathways. TNF-R1 associates with the death-inducing signaling complex (DISC), leading to cleavage and activation of downstream caspases 3 and 7, induce NE apoptosis. In *in vitro* experiments, PKCδ and PI3K can antagonize this effect by inhibiting the interaction between TNF-R1 and the DISC (Kilpatrick et al., 2004; Geering et al., 2022). However, in ALI models, TNF-R inflammatory processes are typically dominated by pro-inflammatory pathways, characterized by increased total NE counts, enhanced exudation, and aggravated lung tissue injury (Zhou et al., 2021; Guo et al., 2024). These dual functional properties render TNF-α a representative biomarker of inflammation in ALI (Raghavendran et al., 2009). At present, most studies have focused on identifying therapeutic agents by inhibiting the TNF-α–NF-κB pathway in LPS-induced ALI models. However, relatively few investigations have explored other TNF-α-associated pro-inflammatory pathways. For example, key downstream mediators of TNF-α signaling, such as IKK and caspase-8 (Loo and Bertrand, 2022), are closely linked to cell death and may serve as indirect drivers of severe inflammation. Validation of these pathways in ALI will require additional mechanistic studies, including gene knockout and pathway-specific intervention experiments. A more comprehensive understanding of TNF-α regulatory mechanisms may facilitate the development of therapeutic agents with greater efficacy and broader pathway coverage.

Mø are also a major source of IL-6. Upon recognition of inflammatory signals by TLRs on M1, IL-6 is rapidly synthesized. When IL-6 acts on NE, it exhibits a dual role similar to that of TNF-α. In its pro-inflammatory role, secreted IL-6 binds to its receptor complex, after associating with the signal-transducing subunit gp130, activates downstream JAK–STAT3 and JAK–SHP2–MAPK signaling pathways. The JAK–STAT3 pathway can further facilitate IL-8 activation and promote NE migration. In addition, IL-6 blockade alone does not affect NE function, suggesting the presence of synergistic cytokines, with STAT3 serving as a key regulatory node in this shared signaling pathway (Fielding et al., 2008; Wright et al., 2014). Experimental studies have demonstrated that targeted inhibition of STAT3 phosphorylation within the IL-6 signaling pathway in ALI mouse models attenuates lung tissue injury and NE infiltration, reduces the secretion of IL-6 and IL-1β, and promotes resolution of inflammation (Cao et al., 2024). Furthermore, numerous therapeutic strategies aimed at inhibiting STAT3 have demonstrated the effectiveness of this anti-inflammatory pathway in ALI (Yang et al., 2021; Zhao et al., 2024).The activity of these pathways is tightly regulated by SOCS3 and SOCS1: SOCS3 binds to phosphorylated gp130 and terminates JAK activation via negative feedback, whereas SOCS1 directly associates with activated JAK to attenuate its activity. In addition, IL-6 receptor activation can directly enhance NF-κB activity and upregulate IL-6 mRNA expression (Kang et al., 2019), a mechanism widely present in innate immune cells such as NE. Conversely, in its anti-inflammatory role, IL-6 reduces NE accumulation by suppressing CXCL-1 and IL-8 levels while upregulating MCP-1 and MCP-2 (Hurst et al., 2001), thereby recruiting Mø and facilitating early apoptosis and clearance of NE (McLoughlin et al., 2003).

IL-1β and IL-18 both belong to the IL-1 family and share approximately 15% sequence homology and highly similar downstream inflammatory signaling pathways. Both cytokines are secreted by M1 and are significantly upregulated in ALI (Xia et al., 2022). Through the MyD88- NF-κB-MAPK pathway (Wawrocki et al., 2016; Yasuda et al., 2019),IL-1β and IL-18 promote NE phagocytosis and recruitment. In ALI models, high-tidal-volume ventilation combined with LPS markedly increases the production of IL-1β and IL-18 by Mø. Among these, IL-1β promotes NET formation and correlates with disease severity in ALI (Nosaka et al., 2025). In a mouse model of blunt chest trauma, knockdown of the PHILDA1 gene suppresses IL-1β levels, indicating a functional association between the two (Wang et al., 2016). Moreover, TLR2 can promote PHILDA1 expression via the JAK2–ERK1/2–STAT3 pathway (Lyu et al., 2016). When TLR2 on the surface of Mø recognizes DAMPs, mediate IL-1β release from Mø. IL-1β subsequently acts on NE to induce NET formation, which in turn functions as a DAMP and amplifies inflammation through a positive feedback loop. However, many regulatory factors involved in this pathway remain unidentified or incompletely characterized. Separately examining the functions and signaling pathways of IL-1β and IL-18 may help identify whether distinct inflammatory mechanisms exist in ALI.

IL-8 is a potent activator of NE and can be produced by Mø, with its levels markedly elevated in ALI (Su et al., 2023). The functional receptors for IL-8 are CXCR1 and CXCR2, which are expressed on NE. Upon ligand binding, IL-8 activatesPI3Kγ and PLCβ2/β3. Among these, PI3Kγ and PLCβ2/β3 regulate NE migration through small GTPases such as Rap1 and Rho. In addition, PLCβ2/β3 can increase IP3 production, thereby activating protein kinase C and mediating the respiratory burst (Matsushima et al., 2022). Furthermore, IL-8 can bind to IgG, forming IL-8–IgG immune complexes, which have been characterized in samples from patients with ALI. Experimental evidence has shown that these complexes bind to FcγRIIa and FcγRIIIb receptors on the surface of NE, broadly activating downstream Src family tyrosine kinases. This process mediates NE recruitment and amplifies inflammation, while also triggering respiratory burst and degranulation, and inhibiting NE apoptosis (Krupa et al., 2004; Fudala et al., 2007). Antibodies against FcγRIIa can suppress the respiratory burst of NE and alleviate oxidative stress-induced injury. Based on these properties, elevated levels of IL-8 immune complexes can serve as indicators of prognosis and mortality in patients with ALI/ARDS (Allen and Kurdowska, 2014). IL-8 is considered one of the most reliable biomarkers for ARDS (Ware et al., 2010), which provides important guidance for assessing disease progression in ALI.

During pulmonary inflammation, M1 secrete large amounts of CXCL-1 and CXCL-2, which act as potent chemoattractants for NE by binding to CXCR2 (Reutershan and Ley, 2004; Zhou et al., 2024), thereby further promoting NE accumulation and exacerbating lung tissue injury. Upon ligand binding, CXCR2 rapidly forms a receptor–ligand complex and activates downstream PI3K–PKB/Akt and phospholipase C-β (PLC-β) signaling pathways, enhancing NE survival, migration, and secretory functions. In addition, CXCR2 signaling can regulate NE rolling and arrest by inducing integrin activation (arrest chemokines). Pharmacological blockade of CXCR1/2 using non-competitive inhibitors has been shown to effectively attenuate lung tissue injury and suppress inflammatory progression (Konrad and Reutershan, 2012).

M2 typically produces protective cytokines, primarily IL-10 and TGF-β. IL-10 is generated following activation of pattern recognition receptors (PRRs) on M2 by inflammatory signals, and its levels are closely associated with the outcome of lung injury (Mahung et al., 2022). IL-10 acts on type II cytokine receptors expressed on NE, mediate anti-inflammatory effects via the JAK1/TYK2–STAT3 pathway (Ouyang and O’Garra, 2019). However, STAT3 is not the only pathway activated by IL-10, and it also serves as a central hub integrating signals from multiple cytokines, so the precise role of IL-10 in inflammation must be interpreted in the context of other signaling pathways and the specific cellular state. Notably, studies have shown that IL-10 levels are elevated in PD-L1-deficient ALI mouse models (Wang et al., 2021). Importantly, IL-10 exerts differential effects on distinct NE subsets. In LPS-induced ALI, IL-10-deficient mice exhibit a marked increase in Fth1^hi^ NE, whereas Prok2^hi^ NE are nearly undetectable in bronchoalveolar lavage fluid (BALF). These findings indicate that Fth1 expression correlates strongly with the severity of lung injury, and the Fth1-to-Prok2 expression ratio may serve as a prognostic biomarker for lung injury (Wang et al., 2022), so identifying the key cellular subsets targeted by IL-10 is valuable for investigation. The protective effects of IL-10 on lung tissue have been supported by extensive experimental evidence (Boehler, 2002; Wu et al., 2009; Lindner et al., 2021). Nevertheless, its clinical translation requires further investigation to establish safety and to ensure maintenance of immune homeostasis.

TGF-β is markedly upregulated in ALI and exerts significant protective effects in lung injury (Pittet et al., 2001; Zhou et al., 2024). It signals through TβRI and TβRII expressed on NE, activating downstream gene transcription and a broad range of signaling pathways via SMAD-dependent and SMAD-independent mechanisms (Mori et al., 2004; Deng et al., 2024). TGF-β suppresses ROS production, degranulation, pro-inflammatory cytokine secretion, and phagocytic activity in NE, while promoting adenosine deaminase activity, thereby accelerating the degradation of injury-induced adenosine. This contributes to reduced NE infiltration and protection of lung tissue (Guven et al., 2013). However, TGF-β can also enhance other NE functions. It inhibit NE migration *in vitro* (Fridlender et al., 2009) and induces phenotypic polarization toward an N2-like state. Additionally, through activating PI3K/Akt or NF-κB to inhibits NE apoptosis (Ear et al., 2010), thereby maintaining an effective population of functional NE at sites of inflammation, which may influence subsequent resolution and tissue repair.

In the field of ALI, cytokines have been extensively studied. Within the context of the NE–Mø crosstalk network, the cytokines discussed in this review primarily function as mediators of intercellular communication, and most studies have focused on the regulatory pathways of individual cytokines. For example, both IL-10 and IL-6 can act synergistically with other cytokines to modulate inflammatory responses. Therefore, further investigation into the interactions among multiple cytokines would contribute to a more comprehensive understanding of the NE–Mø crosstalk network. In addition, the same cytokine may exert distinct effects on different cellular subsets (Wang et al., 2022), highlighting the importance of characterizing cellular heterogeneity in experimental studies. Taken together, for cytokines with dual functions, such as IL-6 and TNF-α, identifying their cellular sources and target cell subsets, as well as determining whether they interact with other cytokines, may facilitate the discovery of more precise regulatory targets and enhance their potential for clinical translation.

### 3.2 Efferocytosis and phagocytosis direct inflammatory outcomes

**Figure 3:**
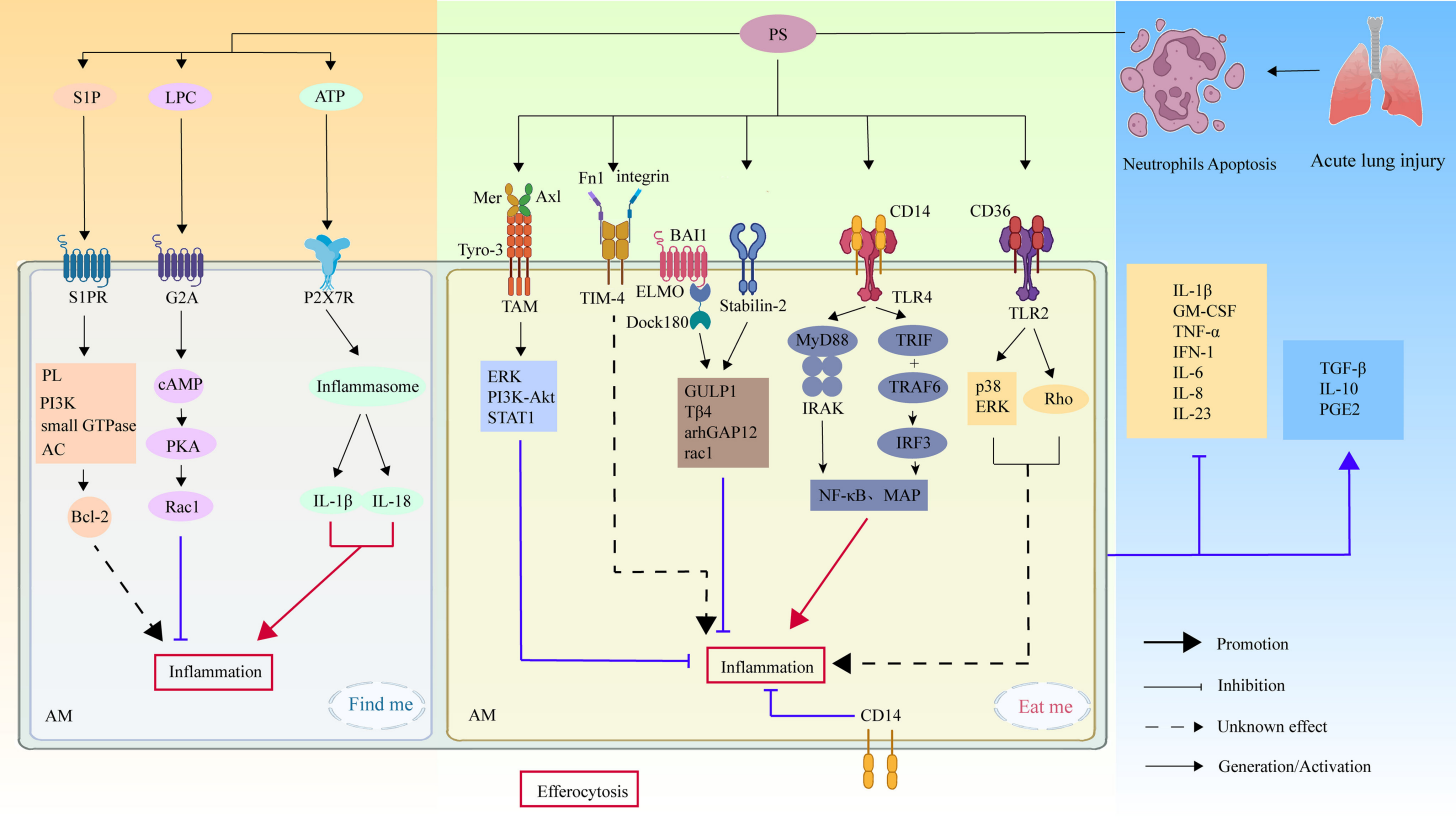
Efferocytosis of apoptotic NE by Mø. Apoptotic NE first release signaling molecules such as S1P, LPC and ATP to attract Mø. Subsequently, phosphatidylserine (PS) exposed on the surface of NE binds to various phagocytic receptors on Mø, thereby activating efferocytosis and downstream metabolic pathways.

In ALI, the efficiency of dead NE clearance may directly determine the trajectory of inflammation, and the metabolic reprogramming that occurs in Mø during phagocytosis can further influence the ultimate inflammatory outcome. The fate of NE in inflammation primarily includes apoptosis and necrosis. Apoptotic NE are cleared by Mø through a process termed “efferocytosis,” which specifically refers to the removal of apoptotic cells by phagocytes. This process also occurs in ALI, and modulation of efferocytosis has been shown to significantly impact disease progression (Leroy et al., 2024; Li et al., 2024a), with tissue-resident Mø (such as AM) playing a predominant role (Zago et al., 2021). Experimental evidence demonstrates that enhancing efferocytosis alleviates inflammation and protects lung tissue (Li et al., 2022), constituting a negative feedback regulatory loop in the inflammatory response (**Figure 3**).

In *in vitro* studies of efferocytosis, M2 exhibits higher efficiency than M1 (Watanabe et al., 2026). Apoptotic NE initially releases “find-me” signals, which include lysophosphatidylcholine (LPC), sphingosine-1-phosphate (S1P), nucleotides (ATP and UTP), and other DAMPs. S1P, generated through phosphorylation by sphingosine kinase (SphK), is secreted and binds to S1P receptors (S1PR) on Mø, activating multiple downstream signaling pathways (Chen et al., 2022). This signaling promotes the expression of Bcl-2 in Mø, thereby prolonging their survival (Weigert et al., 2006). However, the specific role of S1P in ALI appears to vary across different experimental models (Chen et al., 2021; Shi et al., 2021), highlighting the need for further investigation into its underlying regulatory mechanisms. LPC interacts with G2A, activating the cyclic AMP (cAMP)-dependent protein kinase A (PKA) pathway and subsequently Rac1, which drives Mø migration toward apoptotic NE and enhances their phagocytic activity, exerts significant anti-inflammatory effects in ALI (Peter et al., 2008; Frasch et al., 2011; Nan et al., 2022).ATP released during NE apoptosis can act on P2X7R expressed on Mø, activate downstream canonical pro-inflammatory signaling pathways (Virgilio et al., 2017).The therapeutic benefits of blocking this pro-inflammatory pathway have been demonstrated in LPS-induced ALI models (Monção-Ribeiro et al., 2011; Zou et al., 2020). Moreover, elevated P2X7R levels have been associated with poor clinical outcomes in patients with COVID-19. Future studies should validate the role of P2X7R across a broader range of ALI models, as it may serve as a promising prognostic biomarker for predicting clinical outcomes in ALI.

Subsequently, apoptotic NE emit “eat-me” signals that regulate the formation of the phagocytic synapse through the coordinated interaction of phosphatidylserine (PS), bridging protein networks, and phagocytic receptors on the surface of Mø (Ravichandran, 2010). Key receptors involved in the clearance of apoptotic NE include the TAM receptor family, TIM-4, BAI1, Stabilin-2, CD14, CD36, and TLR4 (Parks et al., 2013; Zizzo and Cohen, 2018; Kourtzelis et al., 2020; Su et al., 2021). TAM receptors are critical regulators of M2 polarization, and consist of Tyro-3, Axl, and Mer. TAM receptors indirectly bind phosphatidylserine (PS) through two bridging ligands, growth arrest-specific protein 6 (Gas6) and protein S (ProS). Their downstream signaling pathways are highly diverse and can regulate Mø phagocytosis, anti-apoptotic activity, and secretory functions, while also promoting M2 Mø polarization (Stitt et al., 1995; Myers et al., 2019), M2 Mø mediate the resolution of inflammation in ALI. Among TAM receptors, both PS and ROS generated in the inflammatory milieu can trigger Axl phosphorylation. Dexmedetomidine has been shown to enhance Mø efferocytosis and attenuate ALI inflammation by promoting the ROS/ADAM10/Axl signaling axis (Li et al., 2024a). Mer plays a key role in resolving pulmonary inflammation by facilitating the clearance of apoptotic NE and reducing tissue injury (Ban et al., 2025). Mø lacking Mer retain the ability to recognize and bind apoptotic cells but fail to internalize them. Experimental evidence has shown that ALI leads to a reduction in membrane-anchored Mer levels, accompanied by an increase in soluble Mer. This shift decreases the phagocytic efficiency of Mø, whereas inhibition of this process exerts a protective effect (Lee et al., 2012). In contrast, Tyro-3 has been reported to exert pro-inflammatory effects in platelets and endothelial cells (Laurance et al., 2014; Li et al., 2024b), while contributing to anti-inflammatory outcomes by enhancing Mø phagocytosis; however, its specific role in ALI remains insufficiently characterized.

TIM-4 is expressed on the surface of Mø and specifically recognizes PS via the IgV domain of TIM molecules, directly binding PS to facilitate recognition of apoptotic cells. Studies have shown that TIM-4 requires the formation of a complex with fibronectin (Fn1) and integrins to mediate the engulfment of apoptotic cells (Lee et al., 2019); BAI1, a member of the G protein-coupled receptor family, directly binds PS on the cell surface and forms a tripartite complex with ELMO and Dock180 downstream, activate Rac1 to regulate efferocytosis (D et al., 2007). In ALI mouse models, BAI1 expression is upregulated and has been detected in the nuclei of alveolar Mø, suggesting that its expression or localization may be influenced by pulmonary inflammation (Li et al., 2025). These findings suggest that, in different forms of ALI, the subcellular localization of BAI1 may influence its role in efferocytosis. Stabilin-2 also serves as a direct PS receptor and can activate downstream pathways involving GULP1, thymosin β4 (Tβ4), ArhGAP12, and Rac1, leading to cytoskeletal remodeling (Lee et al., 2008; Park et al., 2008; Bae et al., 2019). This process is closely associated with efferocytosis and promotes the clearance of apoptotic NE by Mø. Additionally, phosphorylation of p38 downstream of Stabilin-2 signaling can induce IL-10 production (Jo and Kim, 2024).

Mø-mediated efferocytosis may be positively correlated with TLR function (Han et al., 2024). Downstream of TLR, two signaling pathways are involved. The MyD88-dependent pathway recruits four IL-1 receptor-associated kinases (IRAKs) to form a complex, leading to early activation of NF-κB and MAPK signaling. In contrast, the TRIF-dependent pathway involves interaction between TRIF and TRAF6, resulting in activation of IRF3 and promotion of type I interferon (IFN) secretion. This process is independent of MyD88 and also contributes to the late-phase activation of NF-κB and MAPK pathways (Kawai and Akira, 2011). In MyD88-deficient mouse models of blunt chest trauma, pulmonary edema, inflammation, and hemorrhage are significantly attenuated, accompanied by markedly reduced levels of IL-6 and CXCL-1 (Hoth et al., 2009), As a shared adaptor in multiple cytokine signaling pathways, MyD88 plays a central role in downstream signal transduction. Investigating its downstream signaling cascades and key regulatory factors may help to further elucidate the mechanisms by which Mø mediate efferocytosis. Additionally, studies have demonstrated that TLR4 expression can influence TAM receptor levels. For example, in ventilator-induced ALI mouse models, knockdown of TLR4 enhances ADAM17-mediated cleavage of the Mer receptor on the surface of Mø, leading to increased levels of soluble Mer. This finding suggests that TLR4 promotes the clearance of apoptotic NE by stabilizing Mer expression on Mø (Su et al., 2021).

CD36, acting as a co-receptor of TLR2, activates downstream p38 and ERK signaling pathways of TLR2 through crosslinking (Erdman et al., 2009). Although this pathway is typically pro-inflammatory, CD36 does not trigger the release of pro-inflammatory cytokines or the activation of the inflammasome during endocytosis. This phenomenon may be associated with the suppressive effects of endocytic processes on inflammatory signaling (Fadok et al., 1998). In addition, co-activation of TLR2 and CD36 by PS leads to activation of Rho GTPases, which mediate actin cytoskeletal rearrangement in Mø and initiate phagocytosis. in the context of lung injury, CD36 has been shown to independently regulate the clearance of apoptotic cells (Parks et al., 2013).

In most studies, CD14 is described as a co-receptor of TLR4, although more recent evidence suggests that TLR4 can function independently of CD14 under certain conditions (Schultz et al., 2025). Taken together, CD14 provides significant accessory support to TLR4 but does not directly regulate its biological effects by modulating endocytosis. In addition, CD14 serves as a direct receptor for apoptotic cells (Devitt et al., 1998), with residue 11 being critical for binding. Furthermore, Li et al. demonstrated that inhibition of CD14 expression can suppress pro-inflammatory signaling pathways, enhance phagocytic activity, and attenuate inflammation in ALI (Li et al., 2021). Notably, the presence of TLR4 appears to inhibit the interaction between CD14 and apoptotic NE (Thomas et al., 2013). While later experimental evidence indicates that CD14-mediated efferocytosis by Mø exerts pronounced anti-inflammatory effects, in contrast to the pro-inflammatory role of CD14 in response to LPS (Thomas et al., 2013).The discrepancy between these findings may be related to the effects of ATRA. ATRA suppresses TLR4-dependent pro-inflammatory signaling while simultaneously reducing CD14 expression, ultimately resulting in an overall anti-inflammatory effect. Consequently, the authors categorized CD14 as part of the pro-inflammatory pathway. However, accumulating evidence from recent studies has gradually distinguished the functions of TLR4 and CD14. CD14 appears to act as a bridge between phagocytes and apoptotic cells, thereby facilitating efferocytosis and exerting anti-inflammatory effects in ALI. Binding of CD14 to apoptotic NE has been reported to promote Mø polarization toward the M2 phenotype, a process further strengthened by crosslinking between CD14 and MerTK (Zizzo and Cohen, 2018),., and its regulatory mechanisms may represent a promising therapeutic target to promote the resolution of pulmonary inflammation and tissue repair.

In *in vitro* and animal studies of ALI, efferocytosis of apoptotic NE appears to promote the transition of Mø toward an anti-inflammatory phenotype. However, in human lung injury, the anti-inflammatory effects of Mø-mediated clearance of apoptotic NE have not yet been definitively established. This discrepancy may be related to the unique alveolar microenvironment, which induces undergo functional and metabolic reprogramming of Mø. Consequently, their interactions with apoptotic NE may differ from those observed in other settings. In addition, NE may not undergo apoptosis in a typical manner (Bersie and McCubbrey, 2025). Studies investigating phagocytic receptors in ALI remain relatively limited, and their downstream regulatory mechanisms still remain to be clarified. This may be attributable to the complex etiology of ALI as well as the extensive crosstalk among different phagocytic receptors, such as TIM-4 and TAM receptors. Moreover, most current evidence is derived from animal models, with relatively little validation available in human studies.

Efferocytosis mediates anti-inflammatory effects by suppressing the secretion of pro-inflammatory cytokines from Mø (Singhal and Kumar, 2022), thereby reducing NE accumulation and functional activity. Additionally, evidence indicates that, in a serum-containing environment, co-culture of apoptotic NE with Mø for 18 hours leads to increased secretion of TGF-β, IL-10, and PGE2 (Greenlee-Wacker, 2016), with IL-10 upregulation showing a time-dependent pattern. Conversely, engulfment of apoptotic NE reduces IL-23 production by Mø, thereby decreasing NE numbers during inflammation via the IL-23/IL-17–G-CSF axis and establishing a closed anti-inflammatory feedback loop (Stark et al., 2005). Inhibition of this axis has shown protective effects in ALI, reducing NE infiltration into lung tissue and attenuating inflammatory injury (Dong et al., 2025). These findings suggest that the IL-23/IL-17–G-CSF axis may represent a promising therapeutic target for the treatment of ALI.

In addition, serum components that promote Mø efferocytosis include β-galactoside-binding lectins, galectin-3, and galectin-1 (Wright et al., 2017; Law et al., 2020). Glycoproteins secreted by Mø, such as MFG-E8 and Gas6, can recognize PS and function as bridging molecules between PS and non-direct receptors. The upregulation of both can exert anti-inflammatory effects in ALI by enhancing efferocytosis (Jeong et al., 2025). Mø can also release the endoplasmic reticulum chaperone calreticulin (CRT), which deposits on the surface of apoptotic NE and serves as an “eat-me” signal to facilitate phagocytosis (Gardai et al., 2005; Feng et al., 2018). In contrast, proteinase 3 (PR-3), expressed as an autoantigen during apoptosis, can antagonize CRT, thereby inhibiting NE clearance (Gabillet et al., 2012).

12/15-lipoxygenase (12/15-LO) can be expressed by specific subsets of Mø and NE (e.g., PMN). In a study by Jiao et al., exosomes derived from M2 (M2-Exos) generated from healthy individuals were administered to ALI mice and were found to exert protective effects. One potential mechanism is the upregulation of 12/15-LO expression, which promotes the conversion of leukotriene B4 (LTB4) into lipoxin A4 (LXA4), thereby facilitating the resolution of inflammation (Jiao et al., 2023). Earlier studies have also demonstrated that LXA4 enhances the efferocytosis of apoptotic NE by Mø. During the engulfment of apoptotic NE, 12/15-LO production is further stimulated, and this process is accompanied by a phenotypic shift of Mø from CD11b^high^ to CD11b^low^ (Godson et al., 2000; Freire-de-Lima et al., 2006). Collectively, these findings suggest that 12/15-LO and LXA4 may form a positive feedback loop that promotes the clearance of apoptotic NE, potentially contributing to the resolution of inflammation in ALI.

The phagocytosis of necrotic NE by Mø does not fall within the scope of efferocytosis; this process is typically referred to as phagocytosis. Unlike efferocytosis, the uptake of necrotic NE leads to upregulation of pro-inflammatory cytokine secretion (Fadok et al., 2001), which may contribute to persistent inflammation and inflammatory amplification in ALI. The phagocytosis of necrotic NE markedly activates pro-inflammatory signaling pathways, with significantly lower induction of TGF-β than that of apoptotic NE, while markedly increasing the production of IL-10, TNF-α, and IL-8. This phenomenon may be associated with the release of immunomodulatory factors from lysed necrotic NE (Pérez-Figueroa et al., 2021). These findings suggest that inflammatory dysregulation in ALI may be related to the level of necrotic NE.

### 3.3 Novel Mø phenotypes in ALI

In recent years, multiple novel AM phenotypes have been identified in bronchoalveolar lavage fluid (BALF) samples from patients and healthy individuals. As resident pulmonary immune cells, AMs are the first to respond to inflammatory stimuli and undergo phenotypic polarization in response to different types of lung inflammation. Investigating their metabolic reprogramming may provide valuable insights into the regulatory mechanisms of ALI. In general, the identification of a new Mø phenotype relies on the characterization of specific markers and cellular products. Based on a review of recent studies, we summarize the Mø phenotypes that may emerge in ALI.

In patients infected with SARS-CoV-2, the lectin CD169 is markedly upregulated in AMs. This specific phenotype enables SARS-CoV-2 entry into AMs in an ACE2-independent manner, and this form of phagocytosis suppresses viral protein expression and virion release (Jalloh et al., 2022). However, CD169+ AMs continuously secrete IL-1β and IL-18, which may promote extensive NET release and NE recruitment, thereby amplifying inflammation and exacerbating lung injury. A study using scRNA-seq of CD169+ Mø identified CD169+ CD11c− AMs as a distinct subset, termed nerve- and airway-associated macrophages (NAMs) (Ural et al., 2020). Notably, in this model, NAMs in poly(I)-induced mice exhibited immunoregulatory functions and prolonged survival. Therefore, CD169+ AMs may represent an important Mø subset involved in ALI-associated inflammatory mechanisms. Further investigation using appropriate experimental models to characterize the intrinsic alterations of this Mø phenotype and its role in inflammation is of considerable value for elucidating disease pathogenesis. In MoAMs, a subset of Mø highly expressing FCGR3B has been identified as being specifically present in severe COVID-19 cases (Nassir et al., 2021). This subtype may be associated with mechanisms of inflammatory dysregulation and may also represent a potential research target within the NE–AM crosstalk network. M2a and M2c Mø are among the most well-characterized Mø subsets in ALI. M2a Mø are characterized by CD206 expression. Bioinformatic analysis and gene knockdown studies have demonstrated that TREM2 acts as an important upstream regulator that mediates M2a polarization via the PI3K/Akt signaling pathway, exerting a protective role in ALI-associated inflammation (Qiao et al., 2023). In contrast, M2c Mø exhibit weaker anti-inflammatory effects than M2a Mø, but they may contribute to pulmonary fibrosis during the late stage of inflammation (Ye et al., 2020). Experimental evidence suggests that IL-10 derived from NE may serve as an important cytokine driving M2c polarization. In addition, TGF-β has been shown to promote Mø polarization toward M2a, M2b, and M2c phenotypes and to attenuate ALI-associated inflammation (Jing et al., 2024). Based on these findings, the IL-10–M2 and TGF-β–M2 axes may represent important therapeutic targets within the NE–Mø crosstalk network. Other Mø phenotypes are largely identified through bioinformatic analyses of BALF samples from patients with ALI, such as Alox15+, PD-L1+, and MIP-1-associated subsets (Morrell et al., 2018; Reyfman et al., 2022; Cao et al., 2023). However, the diverse landscape of Mø phenotypes still requires further validation through experimental (wet-lab) studies.

## 4 The endpoint of the inflammatory circuit—cell death

Cell death is the ultimate outcome of the interaction between Mø and NE, but not the terminal outcome of pulmonary inflammation. It persists throughout ALI and influences ALI outcomes. In addition to mediators within the inflammatory microenvironment of ALI, cytokines or products derived from immunologically active NE and Mø can interact with one another and induce cell death. In recent years, research on cell death has advanced, and multiple distinct modes of cell death have been identified in ALI, including apoptosis, pyroptosis, necroptosis, and PANoptosis. These cell death modalities can occur either between identical or different cell types, such as NE and Mø. Investigating how NE and Mø mutually influence each other to induce cell death, thereby driving either inflammatory resolution or dysregulation, is of great significance for elucidating the pathological mechanisms at different stages of ALI.

In the inflammatory storm of ALI, both NE and Mø undergo apoptosis; According to previous studies (Harris et al., 2019; Wei et al., 2024), NE apoptosis can be detected as early as 6 h after the onset of ALI and increases significantly by 20 h. In contrast, Mø apoptosis occurs mainly by 48 h. However, their apoptotic behaviors appear to exert opposite effects. Promotion of NE apoptosis alleviates inflammation (Zhu et al., 2023), whereas Mø apoptosis contributes to inflammation, and inhibition of Mø apoptosis exerts a protective effect on lung tissue in ALI (Wang et al., 2022; Yang et al., 2023). Cytokines and inflammatory mediators derived from NE and Mø enter the respective cells through secretion or autocrine signaling, thereby activating downstream key regulatory proteins to exert their effects (Matute-Bello et al., 2000; Fan et al., 2022; Youssef et al., 2022; Huang et al., 2025). Among these pathways, PI3K, MAPK, MEK, JAK2, and TNF-α can promote apoptosis by upregulating pro-apoptotic proteins (Bad and Bim) or inhibiting anti-apoptotic proteins (Bcl-2), as validated in ALI models (Ngiam et al., 2007; Yang et al., 2018; Hirata, 2019; Chen et al., 2020; Chen et al., 2021; Shi et al., 2023; Liu et al., 2024). In contrast, FoxO3a promotes SIRT1 transcription and suppresses downstream pro-inflammatory signaling pathways, thereby inhibiting apoptosis of NE and Mø (Wang et al., 2022; Liu et al., 2023). However, complex apoptotic pathways ultimately converge on the activation of caspase-3/7, leading to cellular disassembly. These two key enzymes are closely associated with the survival capacity of NE and Mø. According to relevant studies (Dhlamini et al., 2022; Deng et al., 2026), inhibition of caspase-3/7 may serve as an important therapeutic strategy in ALI by improving AM survival and promoting the resolution of inflammation.

Given the temporal window of apoptosis and the conditions required for its induction, which are largely dependent on the inflammatory environment of ALI, apoptosis of both NE and Mø plays an important regulatory role during the progression of inflammation, contributing either to the resolution of inflammation or to the development of uncontrolled inflammatory responses. During inflammation, NF-κB suppresses intrinsic apoptosis by enhancing Bcl-2 expression, thereby maintaining immune cell survival and preserving normal immune function. however, excessive activation of NF-κB in the late phase of inflammation upregulates RIPK1 and RIPK3, thereby activating a more deleterious pathway—necroptosis.

Necroptosis is induced under strong inflammatory stimulation. Cytokines produced by both NE and Mø, such as TNF and IFN, activate TNFR1 or TLR4, thereby inhibiting Caspase-8 activity and mediating the interaction between RIPK1 and RIPK3 to form the necrosome complex. Notably, TLR downstream signaling can bypass RIPK1 and directly activate RIPK3 (He et al., 2011). Activated RIPK3 phosphorylates MLKL, leading to its oligomerization and subsequent pore formation in the plasma membrane (Alvarez-Diaz et al., 2016). This process results in the substantial release of intracellular DAMPs (e.g., ROS, ATP, IL-1α and pro-IL-1α), among these mediators, pro-IL-1α is biologically active and can be detected as early as 2 h after the onset of inflammation (Dagvadorj et al., 2015). Necrotic alveolar Mø releases it in response to LPS stimulation. Genetic deletion or pharmacological blockade of IL-1α markedly reduces early NE recruitment and pulmonary vascular permeability during the early phase of ALI. In apoptotic cells, IL-1α remains bound to chromatin and does not induce inflammation in this form. However, during necroptosis, IL-1α dissociates from chromatin and is released into the extracellular environment. Among its forms, pro-IL-1α predominantly drives the inflammatory response and regulates subsequent IL-1β release during lung injury (Cohen et al., 2010; Rabolli et al., 2014). Necroptosis involves multiple effector proteins and regulatory enzymes, andthe initiation of necroptotic programs during the crosstalk between NE and Mø is primarily driven by lots of pro-inflammatory factors (Xiao et al., 2025). However, differential release of IL-1α is a hallmark feature that distinguishes necroptosis from other forms of cell death. Therefore, within the NE–Mø crosstalk network, IL-1α released from necrotic AMs can act on IL-1R expressed on the surface of NE, thereby promoting NE recruitment to sites of inflammation. IL-1α and pro-IL-1α play critical roles in the initiation phase of inflammation during ALI, together with other DAMPs, aggravates lung injury (Shotland et al., 2021), thereby promoting both inflammatory escalation and disease progression.

Another cell death pathway, —pyroptosis—has become a major focus in the field due to its involvement in ALI-associated inflammation and its role as a central driver of inflammatory progression (Shen et al., 2024; Zhou et al., 2024). Under physiological conditions, phagocytosis of necrotic or apoptotic NE by Mø contributes to the downregulation of pro-inflammatory mediator levels and facilitates the resolution of inflammation. However, when excessive DAMPs derived from NE act onMø surface receptors (TLR and P2X7R), they activate downstream signaling pathways and trigger pyroptosis. Among these pathways, TLRs activate NF-κB through the MyD88-dependent signaling pathway, laying the foundation for pyroptosis-triggered exacerbation of inflammation (Kinra et al). In contrast, P2X7R triggers the secretion of pro-inflammatory cytokines via caspase-1, a key effector enzyme of pyroptosis.

**Figure 4:**
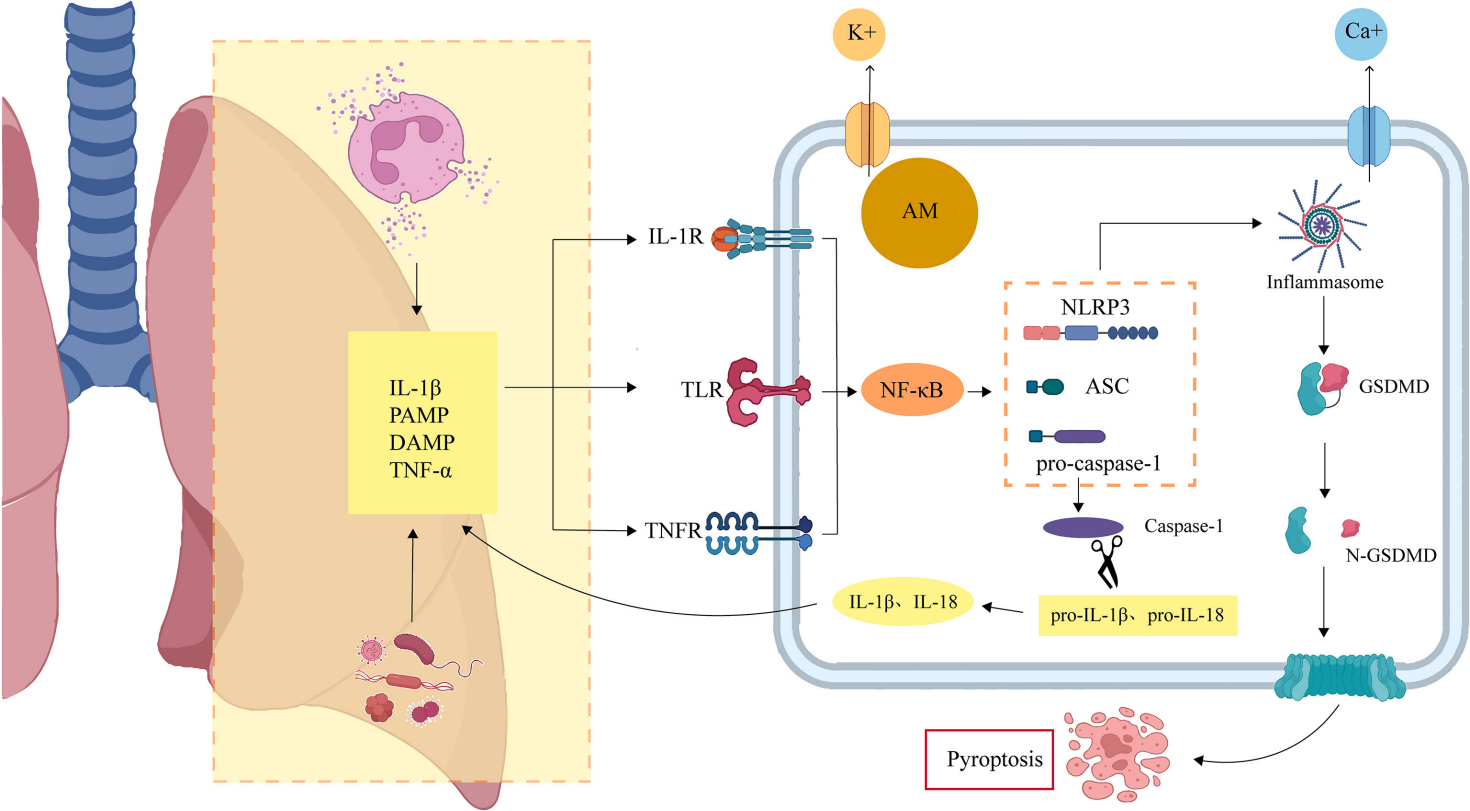
Inflammasome-mediated Mø pyroptosis pathway within the intercellular crosstalk network. IL-1β, TNF-α, and DAMP/PAMPs act on distinct receptors, including IL-1R, TNFR, and TLR, respectively; however, these signals ultimately converge on the NF-κB pathway, initiating inflammasome assembly and promoting pyroptosis. This process leads to the release of additional IL-1β and IL-18, which further recruit NE infiltration and amplify inflammation, thereby forming a positive feedback loop of inflammatory signaling.

These pathways ultimately lead to Mø pyroptosis, resulting in dysregulated and uncontrolled inflammation. The NF-κB pathwaycan further upregulate NLRP3 expression, a key receptor involved in pyroptosis. NLRP3 contains three structural domains—NACHT, LRR, and PYD—and is expressed in both NE and Mø. Unlike membrane-bound receptors, it recognizes intracellular DAMPs, triggering the assembly of the NLRP3 inflammasome and promoting NETosis (Münzer et al., 2021). During inflammation, K^+^ efflux or Ca²^+^ influx can activate NLRP3 in Mø, leading to the release of HSP90 and SGT1 and the recruitment of the adaptor protein ASC and pro-caspase-1. Activated pro-caspase-1 is converted into caspase-1, which processes pro-IL-1β and pro-IL-18, thereby promoting the maturation and secretion of IL-1β and IL-18 (Yin et al., 2023). This process triggers a cytokine storm, and it also cleaves gasdermin D (GSDMD), releasing its N-terminal fragment to induce pyroptosis (Wang et al., 2025), thereby suppressing the ability of Mø to form inflammasomes and impairing their phagocytic function (Son et al., 2021) (**Figure 4**). Because the phagocytic clearance of apoptotic NE by Mø is inhibited, the proportion of M2-polarized Mø is also reduced. Meanwhile, the contents released from dying cells further exacerbate AM death (Luo et al., 2024). This forms a positive feedback loop that amplifies pulmonary inflammation and ultimately leads to exacerbate lung injury. This loop in Mø may be associated with RNA modification (Xu et al., 2021). Among these mechanisms, ADAR1 (adenosine deaminase acting on RNA 1) is highly expressed in AM (Li et al., 2018), but is significantly reduced during lung injury, showing a negative correlation with NLRP3 expression, suggesting that ADAR1 may exert a protective role in ALI. According to previous studies (Zhao et al., 2024), ADAR1 can form an ADAR1/miR-21/A20/NLRP3 cascade in innate immune responses triggered by lung injury, with A20 acting as a central negative regulator, thereby mediating M2 Mø polarization. When tissue injury becomes severe, reduced ADAR1 promotes Mø polarization toward the M1 phenotype. In addition, miR-30d-5p mentioned in Section 2.2 (NE-derived extracellular vesicles) may serve as an independent target to interrupt the inflammatory positive feedback loop centered on M1 polarization in pyroptosis (Jiao et al., 2021).

NE exhibits a marked resistance to these effects, which is associated with two factors. Mitochondrial membrane potential plays an important role in inflammasome-induced cell death, and SARM1, an adaptor protein mediating mitochondrial depolarization downstream of NLRP3 activation (Carty et al., 2019), is expressed in Mø but is negligible in NE. This phenomenon may partly explain NE’s resistance to inflammasome-mediated effects. In addition, caspase-1 levels and GSDMD cleavage capacity are higher in Mø than in NE, resulting in more GSDMD pores on the Mø membrane and thereby promoting pyroptosis. The formation of the inflammasome also promotes the secretion of IL-1β and IL-18 by NE itself, further inducing M1 activation (Zhu et al., 2015; Luo et al., 2024). Collectively, this establishes a pro-inflammatory positive feedback loop centered on the inflammasome with both NE and Mø as target effector cells. Pyroptosis can be detected within hours after the onset of ALI and is characterized by its ability to exacerbate pulmonary inflammation. AM undergo pyroptosis in response to oxidative stress and pro-inflammatory mediators. However, some studies have shown that NLRP3-deficient mice exhibit no significant difference in the extent of NE infiltration during pulmonary inflammation (Dagvadorj et al., 2015; Ikoma et al., 2022), suggesting that pyroptosis has a limited effect on NE recruitment. Therefore, pyroptosis primarily contributes to the amplification phase of the inflammatory cascade in ALI.

AM death represents a critical event in the inflammatory progression of ALI and may constitute a central mechanism driving inflammatory amplification. However, current research on cell death in ALI lacks sufficient translational and clinical validation. Moreover, the extensive crosstalk among distinct cell death pathways adds considerable complexity to mechanistic studies. Future investigations should further define the relevant cellular subsets, stimulatory conditions, and ALI subtypes involved in these processes to facilitate the development of targeted therapeutic strategies. These challenges have also contributed to the emergence of the PANoptosis concept, which provides an integrated framework for understanding the interplay among multiple forms of cell death. ALI caused by various etiologies can trigger PANoptosis through the NE–Mø crosstalk network. During pulmonary inflammation, phagosomes formed through NE–Mø phagocytic activity are degraded in lysosomes, leading to the release of dsDNA, which may originate from bacteria or from dying cells themselves (Underhill and Goodridge, 2012). This dsDNA can bind to surface sensors on NE or Mø and activate the STING–STAT1 pathway, thereby inducing PANoptosis (Messaoud-Nacer et al., 2022). This study also demonstrated that NETs can upregulate key proteins involved in PANoptosis, suggesting that NET formation may further promote PANoptosis. This may represent a central pathway mediating PANoptosis within the NE–Mø network in ALI and may be applicable across multiple forms of ALI. In addition, IFN-β and TNF-α produced during inflammation have also been shown to participate in the induction of PANoptosis in ALI (Karki et al., 2020; Messaoud-Nacer et al., 2022), a process that likewise requires STAT1 involvement, highlighting the critical role of STAT1 in PANoptosis. Furthermore, IL-6, CXCL1, and IL-10 are frequently upregulated during PANoptosis in pulmonary inflammation; however, few cytokines have been shown to independently drive PANoptosis, and they typically act in a synergistic manner. The regulatory relationship between cytokines and PANoptosis still requires further validation using additional models and experimental approaches. These findings, derived from NE- or Mø-associated DNA, NETs, and cytokines, support the persistence of the crosstalk network model under the context of PANoptosis. Both NE and Mø can undergo PANoptosis. NE-associated PANoptosis exerts a dual effect by attenuating inflammation while simultaneously reducing immune competence. NE responds to death signals, releases inflammatory cytokines at early stages, and together with DAMPs released from its own death, acts on Mø. In contrast, Mø responses are relatively delayed; however, the accumulation of downstream pro-inflammatory mediators and amplification of inflammation are primarily driven by Mø.

PANoptosis integrates features of apoptosis, necroptosis, and pyroptosis; however, within PANoptosis, these three forms of cell death do not occur independently. Instead, they are orchestrated by a single multiprotein complex that mediates PANoptosis by recruiting key components from distinct cell death pathways, including RIPK1, RIPK3, CASP8, NLRP3, ASC, and FADD (Christgen et al., 2020). These core components have been validated in PANoptosis signaling pathways, and also play critical roles in ALI (Xiao et al., 2025). In addition, previous hypotheses proposed that these core components function in the form of multiprotein complexes formed through interactions with sensors, collectively termed “PANoptosomes”. RIPK1, ZBP1, and AIM2 are key sensors of PANoptosis, mediating the assembly of three distinct types of PANoptosomes, these PANoptosomes have been molecularly characterized, and both ZBP1-PANoptosomes and AIM2-PANoptosomes have been observed under microscopy (Malireddi et al., 2020; Wang et al., 2022). Collectively, the available studies suggest that these three PANoptosomes may be activated during the development of ALI.

As an important DAMP sensor, RIPK1 is recruited upon TNF-α binding to TNFR. TNF-α may originate from both inflammation-activated Mø and NET-stimulated Mø. Accordingly, RIPK1 activation may represent an important component of the NE–Mø crosstalk network, linking inflammatory signaling to PANoptosis. Through ubiquitination mediated by the E3 ligases c-IAP1/2, RIPK1 recruits downstream caspase-1, ASC, RIPK3, NLRP3, and caspase-8 to form the PANoptosome (Pandeya and Kanneganti, 2023). In contrast, TAK1, which is also linked via ubiquitin chains, can phosphorylate RIPK1 to inhibit its activity, thereby blocking downstream apoptotic signaling and promote inflammation (Geng et al., 2017; Chen et al., 2022). This pathway has been validated in Mø during Yersinia infection (Malireddi et al., 2020). In immunoprecipitation assays, the coordinated changes in related molecules provide evidence for the existence of RIPK1-mediated PANoptosome. This model is primarily driven by the activation of RIPK1-mediated PANoptosome. Experimental results show that RIPK1 knockout mice exhibit reduced numbers of pyroptotic and apoptotic cells, whereas the number of necrotic cells increases. These findings suggest that, although PANoptosis is considered a unified process, different sensor-driven mechanisms lead to distinct patterns of cell death, indicating that functional heterogeneity among PANoptosome is regulated by key modulatory molecules. Although sepsis caused by *Yersinia* infection is often associated with the development of ALI, further validation in a broader range of ALI models is needed to confirm the existence and pathological significance of RIPK1-mediated PANoptosomes in ALI.

The ZBP1-mediated PANoptosome assembly includes the sensor ZBP1, the NLRP3 inflammasome, caspase-8, caspase-6, RIPK1, and RIPK3, among which caspase-6 is a component of the complex formed by ZBP1 and RIPK3 (Zheng et al., 2020), these components are significantly upregulated during pulmonary inflammation. Experimental evidence has shown that TIPE2 expression can suppress ZBP1-mediated PANoptosis, thereby reducing AM death (Wang et al., 2025), this effect is primarily mediated by the downregulation of GSDMD-N and caspase-1 cleavage products downstream of ZBP1-mediated PANoptosis, accompanied by a significant reduction in IL-1β, IL-18, and TNF-α levels. In LPS-induced ALI mouse models, intratracheal administration of lactate was shown to simultaneously downregulate key components of PANoptosis, including ZBP1, caspase-8, MLKL, and GSDMD, thereby suggesting that ZBP1 contributes to lung injury through PANoptosis regulation (Wang et al., 2025). Similarly, stimulation of Mø with CBL0137 resulted in a marked upregulation of ZBP1, further supporting its potential regulatory role in ALI. Unlike the Yersinia-induced sepsis model, ZBP1-mediated PANoptosis in this context is characterized predominantly by pyroptosis and is associated with a pronounced pro-inflammatory effect, suggesting that distinct dominant forms of PANoptosis may be activated under different ALI conditions. In addition, another study investigating caspase-8 in a Yersinia-mimicking model further explored its role in cell death (Muendlein et al., 2021). Immunoprecipitation results demonstrated that ZBP1 and RIPK1 form early complexes in Mø, interacting through RHIM domains, which is a prerequisite for the induction of cell death. The study also established a TRIFosome dependent on the TLR4–TRIF pathway; however, this model has not yet been investigated in ALI. Collectively, these findings support the proposed upstream regulatory role of ZBP1 within the ZBP1-PANoptosome and its early interaction with RIPK1 in driving inflammatory cell death. Assembly of the ZBP1-PANoptosome has been demonstrated in LPS-induced ALI models. However, whether this complex is similarly formed and activated in other ALI models remains to be determined through further experimental validation.

The AIM2-mediated PANoptosome assembly consists of AIM2, Pyrin, ZBP1, ASC, caspase-1, caspase-8, FADD, RIPK1, and RIPK3. In HMGB1-based ALI models, inhibition of AIM2 inflammasome activation significantly reduces the levels of downstream pro-inflammatory cytokines (Wang et al., 2019). Studies have demonstrated that HMGB1 acts on Mø via the TLR2, TLR4, and NF-κB pathways, promoting M1 polarization, while inhibition of TLR2 and TLR4 attenuates AIM2 inflammasome activation, suggesting that TLR2/4 signaling may serve as an upstream regulatory pathway for AIM2 inflammasome formation. Given that HMGB1 can be presented on NETs or released from necrotic cells, and that AIM2 inflammasome activation triggers the secretion of IL-1β and IL-18, NE may exacerbate ALI through the NET–AIM2 inflammasome axis. Furthermore, a study identified a dominant NE subset (CD177+) in sepsis-induced ALI that generates excessive NETs, which may contribute to enhanced inflammation and oxidative stress (Wang et al., 2025). However, these studies have only demonstrated the presence and activation of the AIM2 inflammasome and do not directly establish the existence of AIM2-mediated PANoptosomes in ALI. In ALI induced by long-chain chlorinated paraffins (LCCPs), AIM2 recognizes mitochondrial DNA released from damaged cells and activates downstream signaling, and its upregulation promotes M1 polarization (Deng et al., 2025). This study applied the concept of AIM2-mediated PANoptosome to AMs in pulmonary inflammation; however, experimental validation using ALI-specific models is still lacking to further clarify its precise role in ALI. Taken together, these findings raise the possibility of a positive feedback amplification loop involving NETosis/DAMPs and PANoptosis. Such interactions may contribute to persistent inflammation and progressive lung injury in ALI; however, this hypothesis requires further experimental validation.

**Figure 5:**
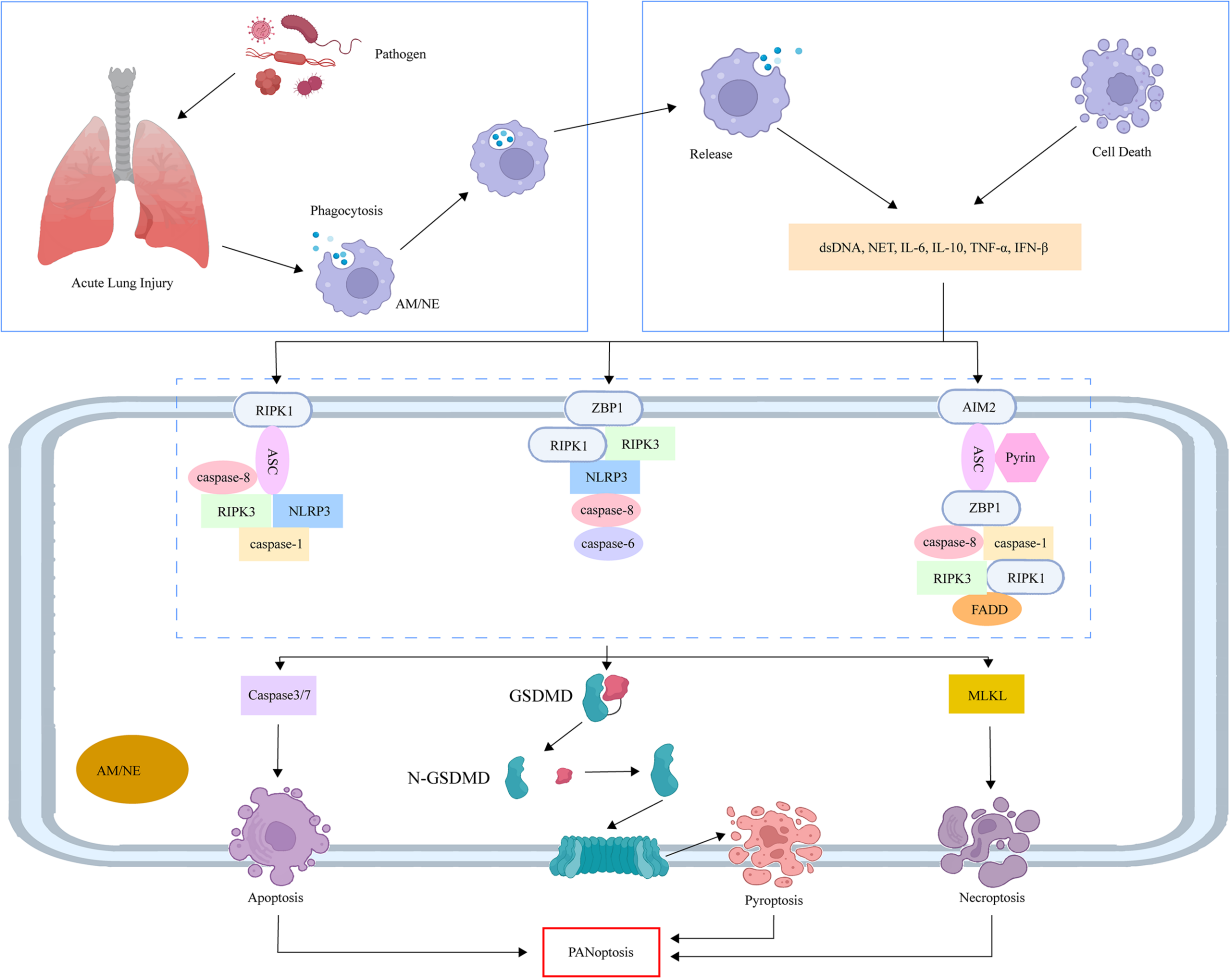
In ALI, NE and Mø undergo PANoptosis triggered by DAMPs. Three types of PANoptosomes can be assembled, and each is capable of mediating three distinct cell death pathways downstream. Consequently, the activation of different PANoptosomes may drive either anti-inflammatory or pro-inflammatory responses.

By simplifying the complex mechanisms underlying PANoptosis, we can summarize that three distinct PANoptosomes are positioned upstream of the pathway, whereas caspase-3/7, GSDMD, and MLKL function as the downstream executioners of three different membrane pore-forming or cell-disassembly processes. These molecules connect PANoptosomes to the execution phase and mediate PANoptosis in ALI through distinct mechanisms. Specifically, GSDMD is responsible for pyroptosis, MLKL mediates necroptosis, and caspase-3/7 executes apoptosis through the orderly dismantling of cells (Briard et al., 2021; Lee et al., 2021; Pandeya and Kanneganti, 2023). (**Figure 5**). The emerging concept of PANoptosis is particularly important in the complex pathogenesis of ALI. It provides a unifying platform that integrates the various forms of cell death involved in ALI, which may help overcome the challenges posed by disease heterogeneity in ALI and holds substantial clinical value and translational potential.

## 5 Translational applications of Ne–Mø crosstalk networks in ALI

Within the NE–Mø crosstalk network, numerous potential targets for clinical translation have been identified, including NETs, EVs, NLRP3, STAT3, and key mediators of PANoptosis. Investigating these targets may facilitate the development of strategies to attenuate inflammatory lung injury, enable earlier diagnosis of ALI, and support the implementation of preventive therapeutic interventions.

The role of mtDNA in ALI has attracted increasing attention in recent years. By triggering NET formation, activating AMs, and promoting inflammatory injury and thrombosis, mtDNA has emerged as an important contributor to lung tissue damage. DNase I efficiently degrades extracellular DNA and has therefore been proposed as a potential therapeutic strategy targeting NETs, with mtDNA representing one of its key substrates. The beneficial effects of DNase I on pulmonary function have been demonstrated in patients with cystic fibrosis (Laube et al., 1996), while its ability to reduce circulating mtDNA and extracellular NETs in ALI has been validated in experimental mouse models (Huang et al., 2023; Jarrahi et al., 2023). In these studies, DNase I was administered either intravenously or via airway inhalation. Airway delivery partially overcomes the limitation of the short systemic half-life of DNase I, although its retention time within the lung may be further optimized through nanotechnology-based delivery systems. Despite these promising findings, several challenges remain before clinical translation can be achieved. First, whether DNase I administration may induce off-target toxicity in other organs remains unclear. Second, excessive degradation of NETs could potentially impair host immune defense and compromise bacterial clearance. Third, the optimal therapeutic window following lung injury has yet to be defined. Furthermore, the experimental models used in these studies do not fully recapitulate infectious ALI. Therefore, additional validation across a broader spectrum of ALI models is required before DNase I can be considered for clinical application. For patients at high risk of transplant-associated ALI, such as those undergoing lung transplantation, the timing of lung injury is predictable (Mallavia et al., 2020). Therefore, DNase I may be particularly suitable for early clinical application in this patient population, where it could potentially serve as a preventive strategy to mitigate inflammatory lung injury. In addition to directly targeting NETs, recent studies have identified methyltransferase-like 3 (METTL3), a key upstream regulator of NET formation (Luo et al., 2026), as a potential therapeutic target. Its inhibitor, STM2457, has shown promise in modulating NET generation at the epigenetic level and may exert broader effects on innate immune signaling pathways. Furthermore, peptidyl arginine deiminase 4 (PAD4), is another critical enzyme involved in NET formation. However, due to the high structural conservation of its catalytic domain, no PAD4 inhibitor with established therapeutic efficacy is currently available (Liu et al., 2021). Interestingly, mesenchymal stem cell (MSC) transplantation has been shown to suppress PAD4 activity and alleviate lung tissue injury in ALI models Interestingly, mesenchymal stem cell (MSC) transplantation has been shown to suppress PAD4 activity and alleviate lung tissue injury in ALI models (Peng et al., 2025). Nevertheless, several challenges remain before MSC-based therapies can be translated into clinical practice, including the evaluation of long-term safety, establishment of standardized dosing regimens, and optimization of delivery methods.

Extracellular vesicles (EVs) have emerged as a major focus of disease diagnosis and therapy in recent years, with their cargo varying according to the cell of origin. In BALF, EVs are predominantly derived from AMs and NEs (Hu et al., 2022), suggesting that their molecular composition may serve as a diagnostic tool for exogenous forms of ALI, such as those induced by pathogen invasion or inhalation of toxic substances. In contrast, serum EVs originate from a broader range of cell types and may therefore be more suitable for identifying endogenous causes of ALI. From a therapeutic perspective, mesenchymal stem cell-derived EVs (MSC-EVs) have been the most extensively investigated. Their ability to suppress M1 Mø polarization and reduce NE accumulation has been demonstrated in multiple animal models of ALI (Deng et al., 2020; Shi et al., 2021). However, EV-based therapies have not yet been translated into clinical practice for ALI. In addition to concerns regarding safety and dosage standardization, the composition of EVs is highly susceptible to environmental influences, resulting in considerable heterogeneity. Furthermore, efficient purification and large-scale production of EVs remain major challenges that must be overcome before their clinical application can be realized.

Most therapeutic studies have focused on upstream regulatory molecules involved in ALI, among which NLRP3 and STAT3 have attracted considerable attention. Thyroid hormones have been shown to improve pulmonary function and attenuate inflammation in ALI (Bhargava et al., 2008). Notably, the thyroid hormone receptor agonist GC-1 can inhibit NLRP3 inflammasome assembly through multiple signaling pathways, thereby reducing Mø pyroptosis and the release of IL-1β and IL-18. These findings suggest a functional interplay between the endocrine and immune systems and indicate that the development of GC-1 may provide a novel perspective for personalized therapeutic approaches in ALI. Similarly, dynasore has been reported to protect lung tissue in ALI through the simultaneous inhibition of the NF-κB pathway and NLRP3 inflammasome activation (Shan et al., 2024). However, its precise regulatory mechanisms and therapeutic efficacy require further investigation. Overall, most current strategies alleviate pulmonary inflammation by suppressing NF-κB signaling and preventing NLRP3 inflammasome assembly. In addition, inhibition of triggering receptor expressed on myeloid cells-1 (TREM-1) suppresses NLRP3 inflammasome activation through metabolic reprogramming, specifically by reducing glycolytic activity in Mø (Zhong et al., 2023). Although the molecular mechanisms linking TREM-1 and NLRP3 remain incompletely understood, the anti-inflammatory effects observed thus far highlight its considerable potential for clinical translation.

STAT3 can be activated by multiple cytokines, including IL-6, IL-10, and members of the IFN family, and its role in ALI has therefore attracted increasing attention. Bioinformatics analyses, network pharmacology studies, and related approaches have suggested that a variety of therapeutic agents exert anti-inflammatory effects through modulation of STAT3 signaling, thereby suppressing downstream inflammatory responses (Liu et al., 2023; Ji et al., 2025; Xu et al., 2025). However, the specific downstream mechanisms and biological consequences of STAT3 activation or inhibition in ALI remain incompletely understood and require validation in animal models. Elucidating these pathways will likely constitute an important focus of future research and may facilitate the development of more targeted therapeutic strategies.

In ALI, therapeutic strategies targeting PANoptosis have primarily focused on several key mediators, including ZBP1, MLKL, and GSDMD. Among these, miR-29a-3p has been shown to simultaneously downregulate the expression of ZBP1, MLKL, and GSDMD in ALI; however, this effect has thus far been demonstrated only in alveolar epithelial cells (Cui et al., 2022), which remains to be validated in AMs and NEs. In addition, KAE attenuates Mø inflammation and cell death by reducing the interaction between RIPK3 and caspase-8 (Chen et al., 2024), although the downstream signaling mechanisms underlying its protective effects warrant further investigation. Bioinformatics analyses have also identified several PANoptosis-associated genes, including NDRG1, DDX3X, PTPRC, and TNFSF8, which are linked to pathways involved in MAPK signaling, phagocytosis, and apoptosis (Lu et al., 2025). Nevertheless, the functional significance of these genes requires further experimental validation. As a relatively emerging area of research in ALI, the translational potential of PANoptosis remains incompletely explored. Future studies should focus on identifying additional regulatory molecules and elucidating the underlying mechanisms governing PANoptosis. Such efforts may expand our understanding of its role in ALI and ultimately establish PANoptosis as a promising therapeutic target for the treatment of this condition.

Due to the marked heterogeneity of ALI, therapeutic targets that demonstrate efficacy in animal models often fail to translate directly into clinical benefit in humans. This represents one of the major obstacles to successful clinical translation. To address this challenge, porcine models have increasingly been employed in ALI research because of their close anatomical and physiological similarities to humans. Notably, a unilateral porcine ALI model has been shown to reproduce several key features of clinical ARDS, including severe alveolar epithelial injury, reduced lung compliance, hemodynamic stability, and metabolic stability, making it more representative of human disease than conventional LPS-induced or lung lavage models (Geilen et al., 2022). Therefore, the development of more clinically relevant ALI models is likely to become an important focus of future research.

## 6 Conclusion and perspectives

This review summarizes how NE and Mø interact in the context of ALI. The inflammatory process is systematically divided into three components: secretion, efferocytosis, and cell death, with the associated regulatory pathways comprehensively discussed. By constructing an inflammatory network based on NE–Mø crosstalk and further elucidating the underlying mechanisms of ALI, future research may pave the way for a transition from symptom-oriented management to etiology-driven therapeutic interventions.

With the progression of inflammation, the interaction between NE and Mø becomes increasingly complex. Early recognition of ALI and timely intervention can improve prognosis. Therefore, certain cytokines, due to their temporal sensitivity and responsiveness, have already been applied as biomarkers of lung injury in clinical practice (Gonzales et al., 2015). However, therapeutic strategies directly targeting cytokines remain limited. Many studies have focused on upstream regulators of pro-inflammatory cytokines in an attempt to identify therapeutic targets that reduce inflammation and protect lung tissue. Nevertheless, a single cytokine may exert different functions across ALI models, form complexes, or display dual roles, which complicates the interpretation of its regulatory pathways. Furthermore, whether inhibition of pro-inflammatory cytokines may exacerbate infection in ALI or disrupt immune homeostasis remains an important clinical concern that requires careful consideration.

At present, Mø polarization in ALI research is still mainly classified into M1 and M2 phenotypes. However, accumulating evidence has demonstrated that further refinement of Mø phenotypes and functional validation are of considerable value for elucidating regulatory mechanisms. AMs are an important component in the pathogenesis of ALI, and the nature of inflammatory stimuli (such as bacteria, viruses, or cytokines) can significantly influence their polarization states. However, most experimental studies rely on LPS-induced ALI models, which may lead to a relatively homogeneous AM phenotype and limit in-depth investigation of regulatory mechanisms. Therefore, developing more specific and physiologically relevant ALI models remains an important challenge for the future. Improved modeling strategies may enhance the translational potential of mechanistic studies and facilitate the transition from basic research to clinical application (Zhang et al., 2024).

Cell death represents a central axis within the NE–Mø crosstalk network. By separately examining apoptosis, necroptosis, and pyroptosis, a tentative “timeline” of ALI can be outlined. During the early stage of inflammation, AM undergo necroptosis and release pro-IL-1α/IL-1α and other signaling molecules, thereby recruiting NE and initiating the inflammatory response. As the inflammatory milieu evolves, apoptosis becomes an important regulatory mechanism, directing the progression of ALI toward divergent outcomes. Pyroptosis, in contrast, may represent an important contributor to inflammatory amplification during the cascade phase. These forms of cell death are interconnected and mutually influential, collectively constituting the process of PANoptosis. Nevertheless, it remains unclear whether distinct forms of cell death occur in a temporally coordinated manner and how they contribute to different stages of ALI progression. Addressing these questions will require further mechanistic and temporal studies. Current studies on PANoptosis in the field of ALI remain at the hypothesis-validation stage partly. Whether many PANoptosomes that have been well characterized in other disease settings are similarly activated during ALI-associated inflammation? That requires further investigation. Moreover, the predominant mode of pore formation may influence the outcome of the inflammation. The conceptual framework of PANoptosis facilitates a systematic and dynamic understanding of the diverse forms of cell death occurring in ALI, enables the identification of shared regulatory targets, and may provide valuable insights for the development of etiology-based therapeutic strategies for ALI.

From the perspective of this review, integrating the NE–Mø crosstalk network into the pathological framework of ALI can render the inflammatory cascade more temporally ordered and systematically integrated. This approach extends the characterization of ALI beyond “alveolar epithelial injury and impaired respiratory function” to a more in-depth understanding of specific inflammatory regulatory mechanisms. In the future, emerging technologies, including single-cell transcriptomics, spatial transcriptomics, and multi-omics approaches, may facilitate the identification of additional cellular mediators within this crosstalk. These approaches may also help to systematize complex signaling pathways and support the development of more comprehensive ALI models. Such advances may support the development of more comprehensive therapeutic strategies for ALI. This could facilitate stage-specific and mechanism-based interventions across different phases of inflammation.

## Glossary

ALI: Acute lung injury
ARDS: Acute respiratory distress syndrome
AM: Alveolar macrophage
ARG1: Arginase-1
JNK: c-Jun N-terminal kinase
CSS: Cytokine storm syndrome
DAMPs: Damage-associated molecular patterns
CTSC: Cathepsin C
ERK: Extracellular signal-regulated kinase
EVs: Extracellular vesicles
GSDMD: Gasdermin D
GPCRs: G protein-coupled receptors
HMGB1: High-mobility group box 1
HIF-1α: Hypoxia-inducible factor-1α
iNOS: Inducible nitric oxide synthase
IL-1β: Interleukin-1β
IL-6: Interleukin-6
IL-8: Interleukin-8
IL-10: Interleukin-10
IL-18: Interleukin-18
IM: Interstitial macrophage
LTB4: Leukotriene B4
LGICs: Ligand-gated ion channels
LPS: Lipopolysaccharide
MIF: Macrophage migration inhibitory factor
Mø: Macrophages
mPR3: Membrane serine protease 3
MAPK: Mitogen-activated protein kinase
MLKL: Mixed lineage kinase domain-like protein
Mo-AM: Monocyte-derived AM
MyD88: Myeloid differentiation primary response 88
MPO: Myeloperoxidase
NDMVs: NE-derived microvesicles
NETs: Neutrophil extracellular traps
ENDS: Elongated neutrophil-derived structures
NDTRs: Neutrophil-derived trails
NE: Neutrophils
NO: Nitric oxide
NLRP3: NOD-like receptor family pyrin domain containing 3
NF-κB: Nuclear factor-κB
OXPHOS: Oxidative phosphorylation
P2X7R: P2X7 receptor
PANoptosis: PANoptosis
PAMP: Pathogen-associated molecular pattern
PPARγ: Peroxisome proliferator-activated receptor γ
PGC-1β: Peroxisome proliferator-activated receptor gamma coactivator-1β
PMA: Phorbol 12-myristate 13-acetate
PS: Phosphatidylserine
PM: Pulmonary macrophages
ROI: Reactive oxygen intermediates
ROS: Reactive oxygen species
RAGE: Receptor for advanced glycation end products
RTKs: Receptor tyrosine kinases
STAT1: Signal transducer and activator of transcription 1
STAT3: Signal transducer and activator of transcription 3
SIRT1: Sirtuin 1
S1P: Sphingosine-1-phosphate
STING: Stimulator of interferon genes
SOCS-1: Suppressor of cytokine signaling 1
TRIF: TIR-domain-containing adapter-inducing interferon-β
TR-AM: Tissue-resident AM
TIR: Toll/interleukin-1 receptor
TLR4: Toll-like receptor 4
TLRs: Toll-like receptors
TGF-β: Transforming growth factor-β
TREM-1: Triggering receptor expressed on myeloid cells-1
TCA: Tricarboxylic acid
TNF-α: Tumor necrosis factor-α
ZBP1: Z-DNA-binding protein 1
ZymA: Zymosan A
